# Supplementary material for: A multiscale screening platform for rapid GPCR variant profiling reveals color-tuned JSR1 mutants
Source: Biophys J. 2025 Oct 27;125(10):2272–86. doi: 10.1016/j.bpj.2025.10.034 (PMC13351981; doi:10.1016/j.bpj.2025.10.034)
Supplement: Document S1. Figures S1–S9 [file mmc1.pdf]

Biophysical Journal, Volume *125*

Supplemental information

A multiscale screening platform for rapid GPCR variant profiling reveals color-tuned JSR1 mutants

Jonas Mühle, Deborah Walter, Marielouise Griebl, and Gebhard F.X. Schertler

A multiscale screening platform for rapid GPCR variant profiling reveals color-tuned JSR1 mutants

Supplemental material

Jonas Mühle[1,*], Deborah Walter[1,2], Marielouise Griebl[1,§], Gebhard F. X. Schertler[1,*]

1 Center for Life Sciences, Laboratory of Biomolecular Research, Paul Scherrer Institute, Villigen PSI, Switzerland.

2 Biomolecular Structure and Mechanism PhD Program of the Life Science Zurich Graduate School, University of Zurich, Switzerland

§ Current address: Molecular Biotechnology Master Program, Faculty for Engineering, Heidelberg University, Germany

* correspondence to: jonas.muehle@psi.ch and gebhard.schertler@psi.ch

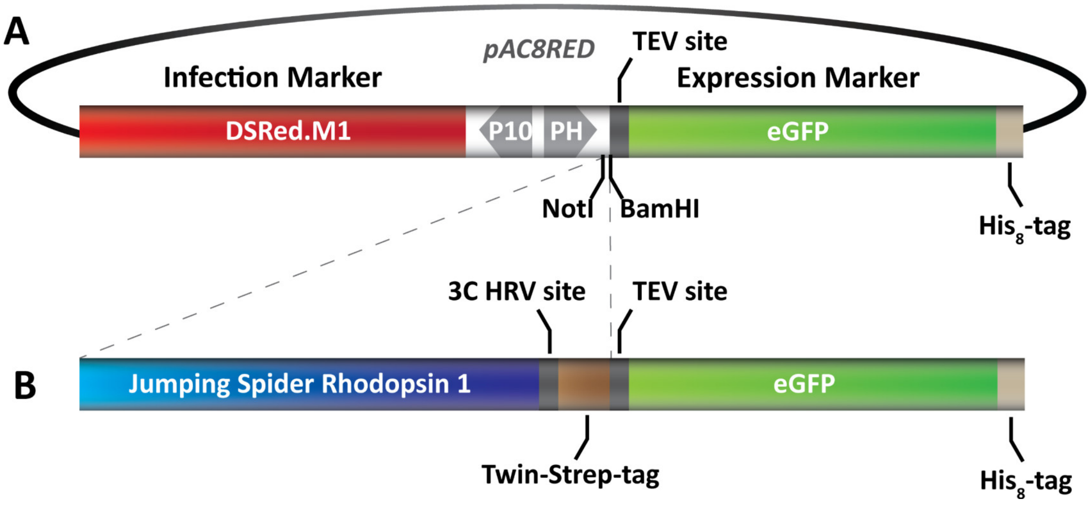

Supplemental Figure S1: Plasmid and construct design of the JSR1-eGFP fusions. A) *pAC8RED backbone with two fluorescent proteins. DsRed.M1 under the P10 promoter control used as an infection marker and TEV-eGFP-His$_8$ sequence under the PH promoter control. NotI and BamHI restriction sites are used to insert covalent fusions of the target proteins N-terminally to the TEV-eGFP-His$_8$ cassette.* ***B)*** *Jumping Spider Rhodopsin 1 (JSR1) inserted via the NotI/BamHI sites. A 3C HRC protease sequence followed by a Twin-Strep-tag was fused C-terminally to JSR1.*

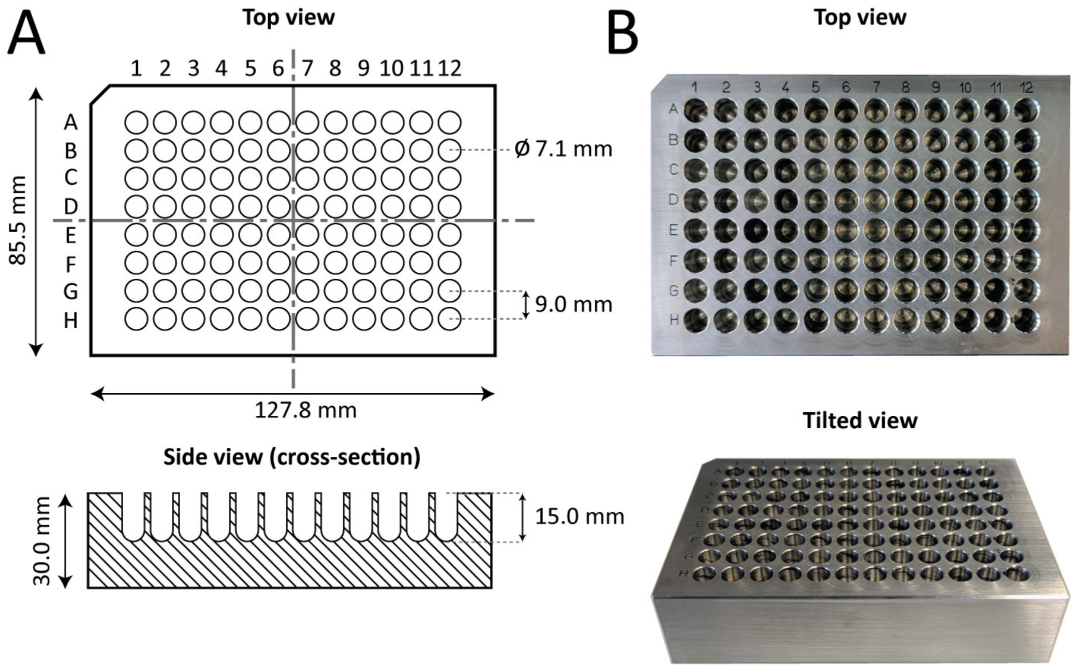

***Supplemental Figure S2: Blueprint and images of the custom-made ultracentrifuge tube holder for Beckmann Type 42.2 Ti tubes**. **A)** Top view and side view blueprint of the tube holder. The holder was manufactured from a solid aluminum block according to standard 96-well microplate dimensions defined by ANSI/SLAS (https://www.slas.org/SLAS/assets/File/public/standards/ANSI_SLAS_1-2004_FootprintDimensions.pdf). For efficient temperature control, the plate height is 30.0 mm and the holes are 15.0 mm deep with a 7.1 mm diameter. **B)** Top view and tilted view images of the tube holder. The metal block was not built for the use in any centrifuge or ultracentrifuge. It does not hold 96-well plates or enables high-speed centrifugations of those. It solely provides an efficient opportunity to transfer liquids from 96-well plates into single ultracentrifuge tubes and vice versa using multichannel pipets before and after ultracentrifugation. It helps reduce pipetting errors or mix-up of samples (particularly in dim-light conditions) while keeping the samples cold (or warm, if desired).*

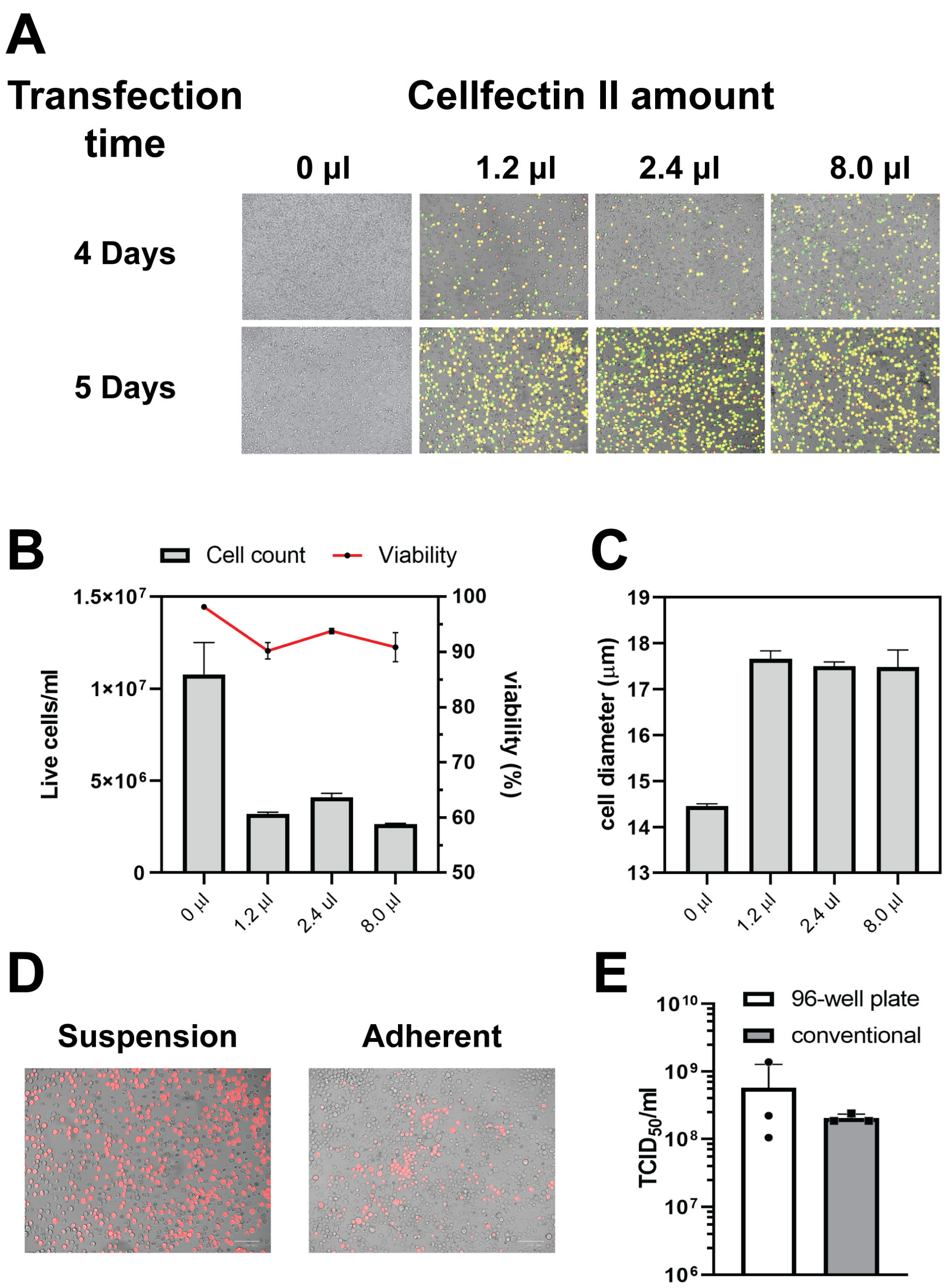

Supplemental Figure S3**: **Transfection and virus amplification in suspension. A) *Transfection efficiency monitored by fluorescence microscopy after 4 and 5 days post-transfection at different Cellfectin II concentrations.* ***B) and C)*** *Cell fitness parameters (cell density, viability, and diameter) were assessed 4 days post-transfection. The sample names correspond to the Cellfectin II amounts used for transfection. All transfected cells show clear signs of a viral infection compared to the negative control ("0 µl"): reduced viability, reduced cell growth, and increased cell diameter. No significant differences between the three transfected samples are observed. Values are derived from two independent replicates, displayed ± S.D.* ***D)*** *Comparison of transfection in suspension (in 96-Well DWPs) with conventional protocols in adherent cell culture (in 6 well plates) monitored by fluorescence microscopy 5 days post-transfection.* ***E)*** *Assessment of viral strength after one cycle of virus amplification (V_1 virus stocks) in suspension cultures using a TCID50 assay. Both methods produce virus stocks with very similar viral strengths. Values represent the mean ± S.D. of three independent replicates.*

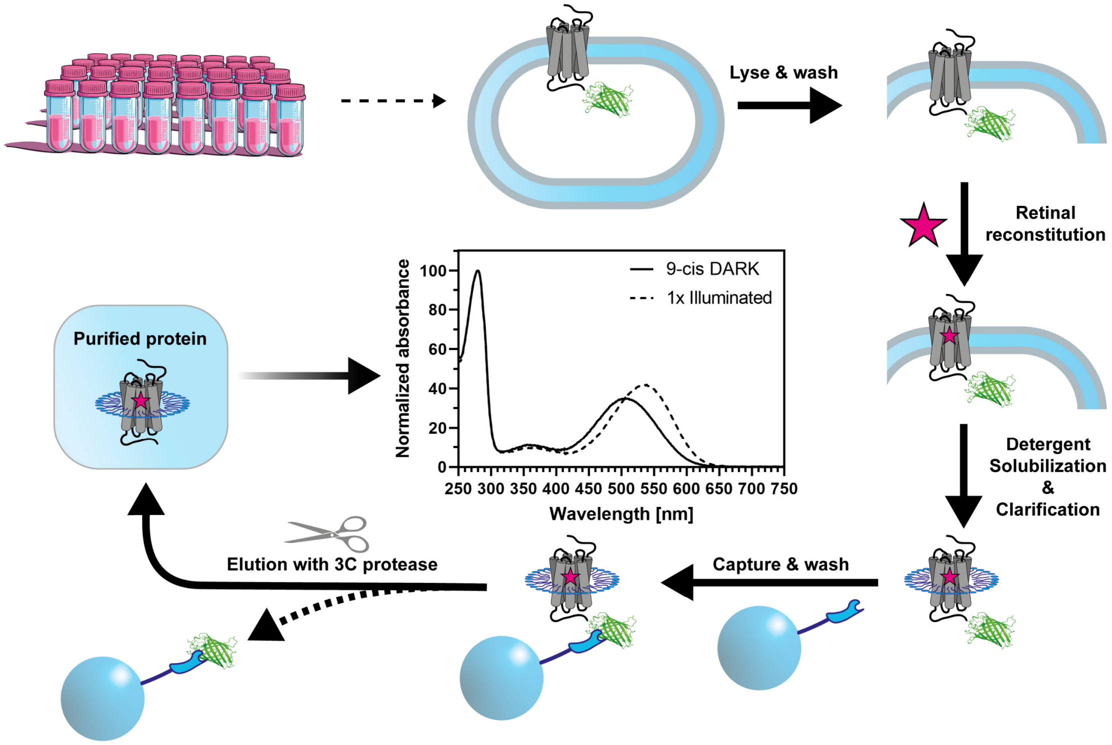

***Supplemental Figure S4**: **Workflow from 25 ml expression cultures to UV/Vis absorption analysis** of small-scale purified proteins. The proteins were expressed in TPP Tubespin Bioreactors 50. The pellets were lysed, and the insoluble fraction was washed before JSR1 reconstitution with retinal. The detergent-solubilized receptor was captured by anti-eGFP- or StrepTactin XT resin, washed, and eluted with 3C protease, which yielded highly pure, fluorophore-free JSR1 variants for the characterization of the UV/Vis absorption properties.*

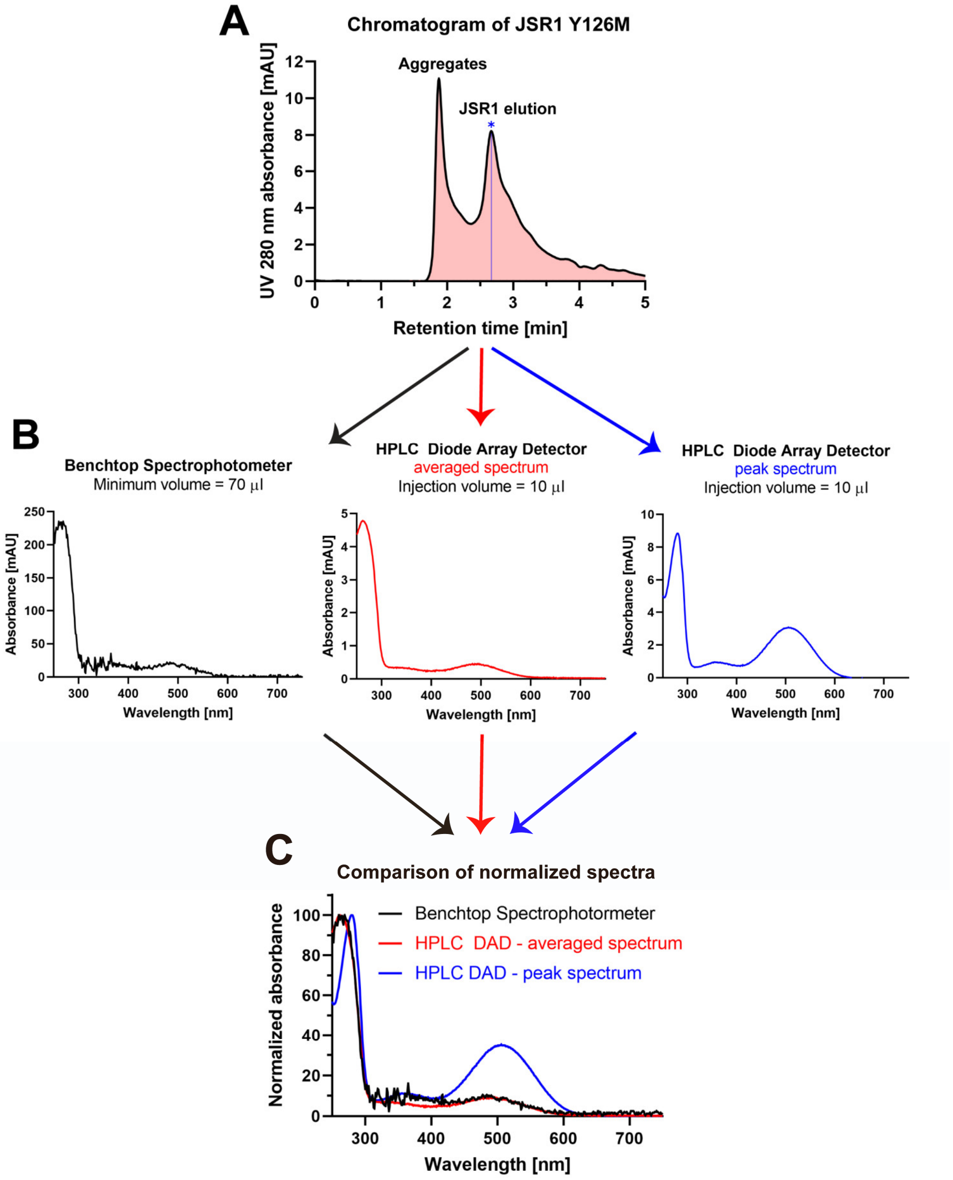

Supplemental Figure S5: Comparison of UV/Vis absorption spectra recorded on a benchtop spectrophotometer and HPLC Diode Array Detector (DAD). A) *Chromatogram of affinity-purified JSR1 Y126M. The sample contained a significant carryover of aggregates and was eluted in a total of 300 µl elution buffer due to technical complications during the small-scale purification procedure. The sample represents the most impure sample of the entire set of mutants and was used to compare the data quality of different absorption spectra acquisition methods.* ***B)*** *UV/Vis absorption spectra acquired from the purified sample. Left: Using a standard benchtop spectrophotometer and a cuvette with 10 mm pathlength. The spectrum displays a large peak at 260/280 nm, a noisy region between 300-400 nm, and a small chromophore absorption peak around 500 nm. Center: Spectrum retrieved from the HPLC DAD detector by averaging all spectra across the entire size exclusion chromatography run (red area in the chromatogram above). This spectrum simulates the spectrum from the benchtop spectrophotometer as it contains all components from the affinity purification without further size exclusion chromatography purification. Despite a 50 times lower signal intensity, the spectrum displays much less noise. Right: Peak spectrum extracted from the peak maximum of the JSR1 Y126M elution (indicated with a blue line and an asterisk in the chromatogram). The spectrum shows a clear peak at 280 nm and a strong chromophore absorption peak around 500 nm.* ***C)*** *Overlay of the three spectra after normalization to the global peak maximum. The averaged DAD spectrum is indeed highly similar to the benchtop spectrophotometer spectrum. However, the DAD provides a substantially improved signal stability across the entire spectral range. The DAD peak spectrum has a clearer 280 nm peak and a significantly increased 280 nm/ 500 nm ratio compared to the other spectra. This comparison highlights the power of an HPLC-DAD-based UV/Vis analysis over conventional methods. It drastically enhances the confidence of the analysis and eliminates the need for larger quantities of highly purified proteins.*

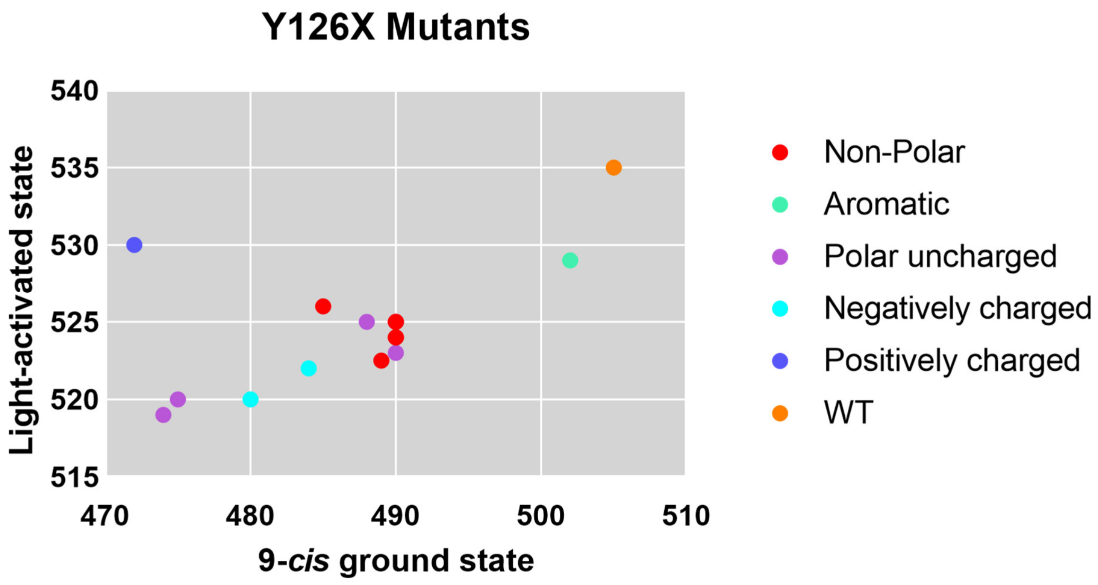

***Supplemental Figure S6**: **Analysis of λ_{max} of all Y126X mutants in the 9-cis dark state against the activated state**. The WT (Y126) is the most red-shifted variant at that position in both states. The overall most similar mutant in both states is Y126F (the only aromatic mutant that expressed). Y126H (the only positively charged mutant that expressed) induces the largest blue shift in the 9-cis dark state while retaining the most similar absorption to the WT in the active state, leading to an enlarged spectral separation of the two states.*

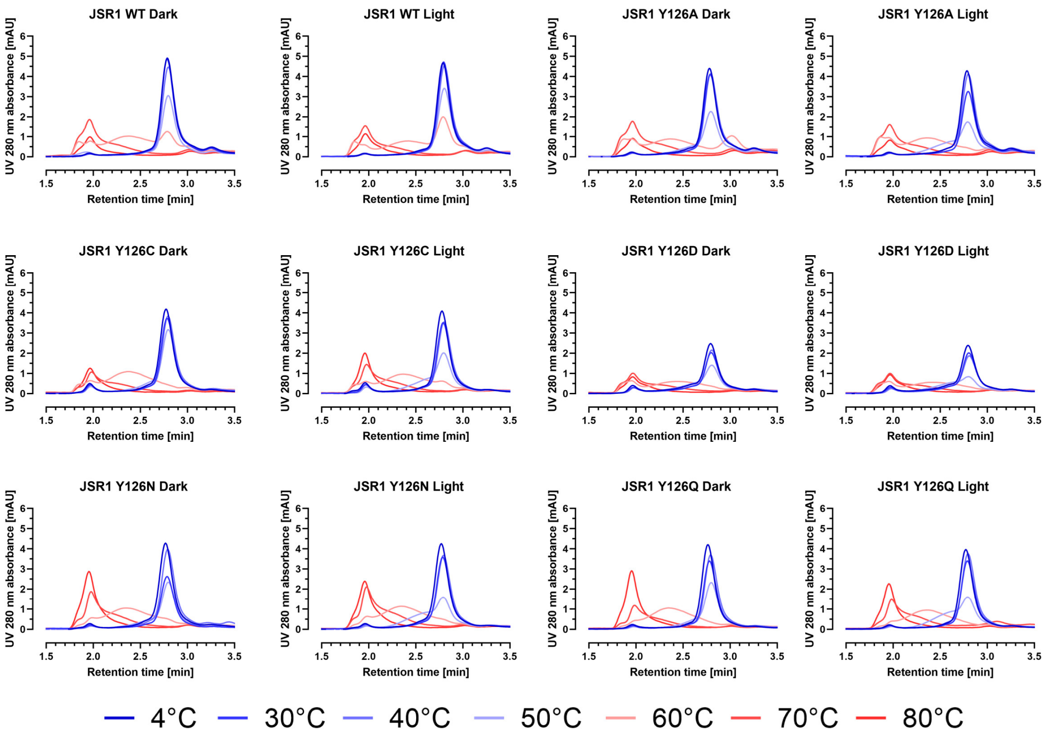

***Supplemental Figure S7**: **Analytical Size Exclusion chromatograms (UV$_{280nm}$) used to assess the thermal stability of JSR1 WT and five Y126X mutants**. Each panel displays all chromatograms from a single JSR1 variant in either the ground state ("Dark") or the light-activated state ("Light"). The samples were incubated for 5 minutes at different temperatures prior to the SEC analysis, and the chromatograms are colored according to the temperature gradient from blue (4°C) to red (80°C). UV/Vis spectra were extracted at the monomeric peak position of JSR1 at 2.8 minutes and plotted in Supplemental Figure S8.*

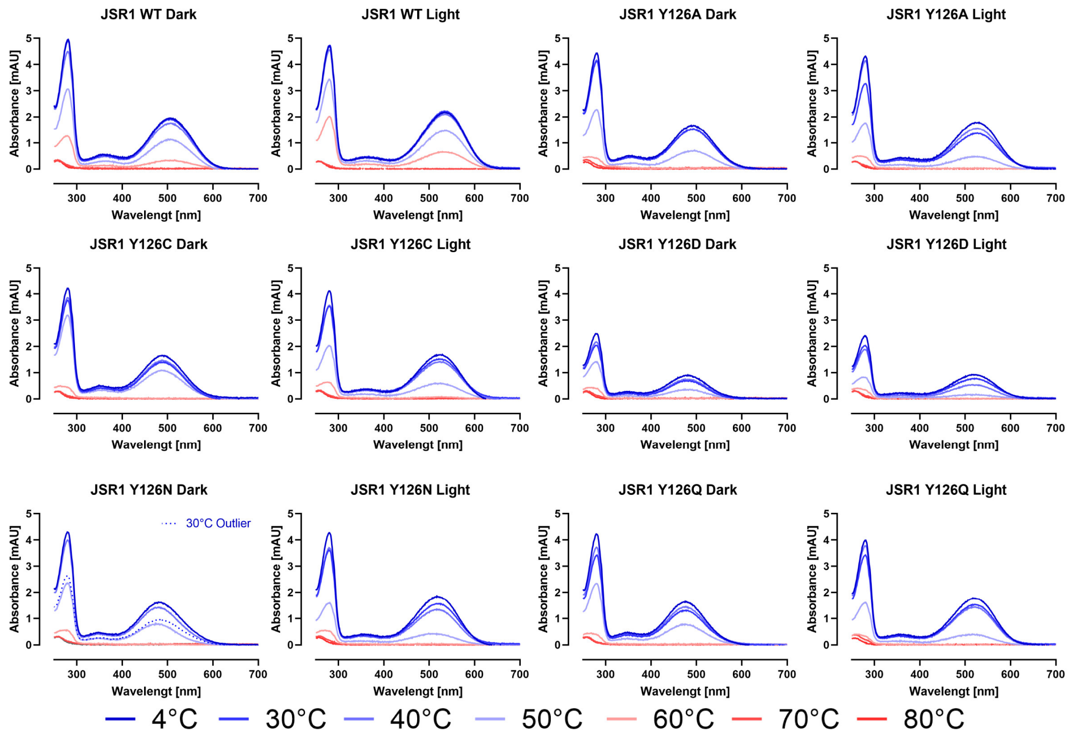

***Supplemental Figure S8**: **UV/Vis spectra extracted at 2.8 min from the UV_{280nm} chromatograms in Supplemental Figure S7**. Each panel displays all spectra from a single JSR1 variant in either the ground state ("Dark") or the light-activated state ("Light"). The samples were incubated for 5 minutes at different temperatures prior to the SEC analysis, and the spectra are colored according to the temperature gradient from blue (4°C) to red (80°C). We used the thermal degradation of the chromophore absorption peak to determine the melting temperatures (see Fig. S9).*

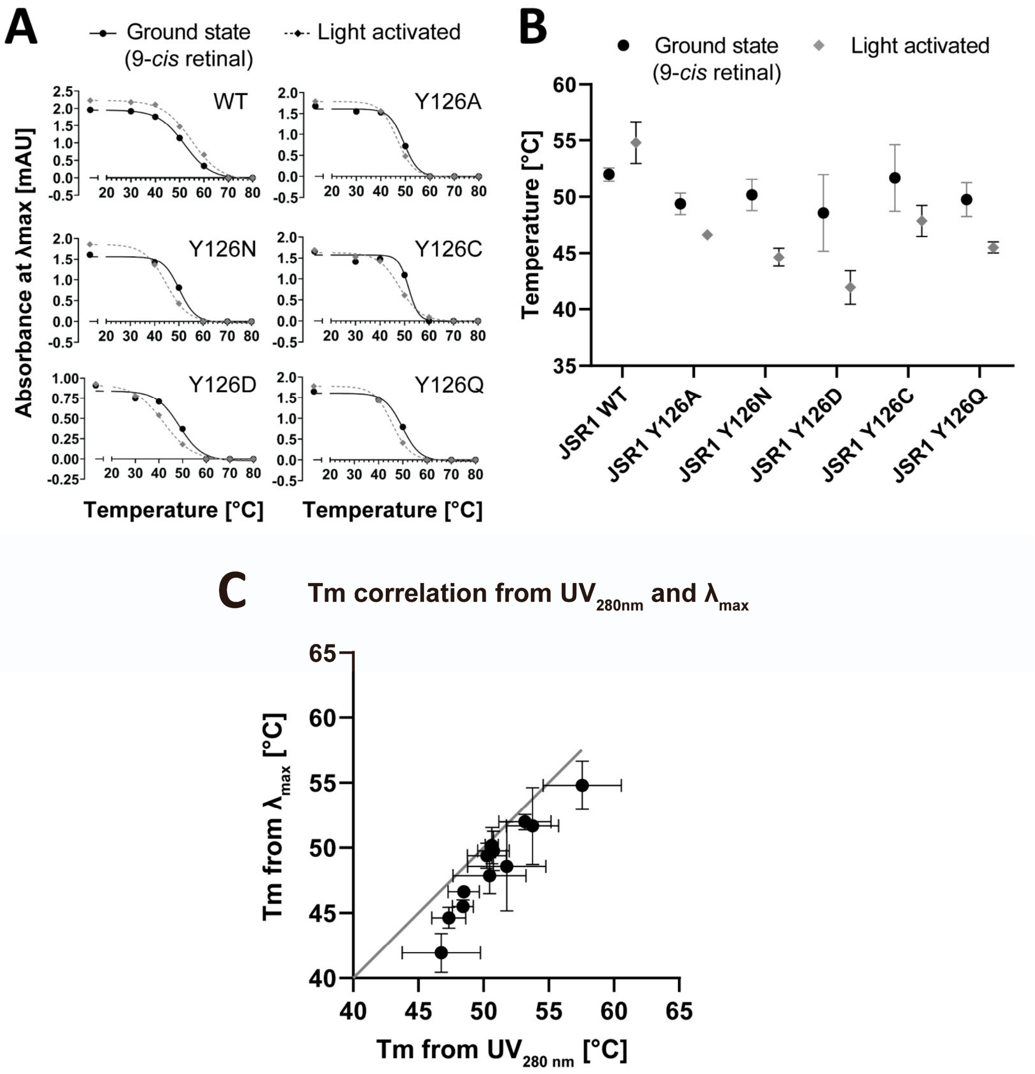

Supplemental Figure S9*:** **Thermostability data derived from the chromophore absorption. A)** *HPLC-derived melting curves of different JSR1 mutants obtained from the decay of the chromophore absorption at increasing temperatures (see Suppl. Fig. S8). The data represent the peak height of the chromophore absorption peak at the corresponding temperature. Values from the ground state proteins are shown as black dots, while data after light activation are shown as grey rhombuses. The data was fitted by a Boltzmann sigmoidal equation. Fitted curves are shown as black solid (ground state) and grey dashed (active state) lines.* ***B) *Melting temperatures calculated from the melting curves. The melting points are shown as black dots (ground state) and grey rhombuses (light-activated state). Error bars indicate the upper and lower limits of the 95% confidence interval of the fit.* ***C)*** *Scatter plot showing the correlation between the melting temperature (Tm) calculations from the UV_{280nm} signal (plotted on the X-axis) vs the λ_{max} of the JSR1 chromophore (plotted on the Y-axis). The data is shown as black dots with their corresponding upper and lower limits of the 95% confidence interval of the fit. Each point represents one sample. A diagonal through the origin with slope = 1 is shown as a grey line representing the theoretical perfect correlation. For most of the samples, this diagonal is within the 95% confidence interval, demonstrating a good agreement between both methods. However, a small systematic bias towards higher melting temperatures from the UV_{280nm} data is observed.*
