# Supplementary material for: A multiscale screening platform for rapid GPCR variant profiling reveals color-tuned JSR1 mutants
Source: Biophys J. 2025 Oct 27;125(10):2272–86. doi: 10.1016/j.bpj.2025.10.034 (PMC13351981; doi:10.1016/j.bpj.2025.10.034)
Supplement: Document S2. Article plus supporting material [file mmc2.pdf]

Biophysical Journal
Article

A multiscale screening platform for rapid GPCR variant profiling reveals color-tuned JSR1 mutants

Jonas Mühle,[1,*] Deborah Walter,[1,2] Marielouise Griebl,[1] and Gebhard F. X. Schertler[1,*]
[1]Center for Life Sciences, Laboratory of Biomolecular Research, Paul Scherrer Institute, Villigen PSI, Switzerland and [2]Biomolecular Structure and Mechanism PhD Program of the Life Science Zurich Graduate School, University of Zurich, Zurich, Switzerland

ABSTRACT Integral membrane proteins such as G-protein-coupled receptors (GPCRs), including visual pigments, are critical targets in both fundamental and pharmaceutical research. However, low expression levels and intrinsic instability often hinder their functional and structural characterization. Therefore, efficient screening methods are needed to identify suitable candidates for biochemical and biophysical analysis. Although the insect cell baculovirus expression system is commonly used for the production of functional GPCRs, the virus generation steps are labor intensive, hampering efficient high-throughput screening. In this study, we addressed this limitation by developing a platform for rapid high-titer baculovirus generation in 96-well suspension cultures and subsequent protein expression screening using the same format. The strength of our platform lies in its modular, multiscale design. Beyond the 96-well micro-scale screening phase, we provide protocols for expression of selected variants in 25-mL cultures, followed by a mini-scale affinity purification. The protein yields are sufficient for functional characterization of multiple GPCR variants in parallel using diverse in vitro assays. This intermediate screening step enables data-driven selection of promising candidates for large-scale expression and further characterization. We demonstrate the platform's capabilities through a mutant screen of the light-sensitive GPCR Jumping Spider Rhodopsin 1 (JSR1). From 56 JSR1 mutants, we selected 30 variants for further characterization after expression screening. For all these, we obtained high-quality UV/Vis absorption spectra in both inactive and active states. Remarkably, we discovered five previously uncharacterized mutants with well-separated absorption maxima that are promising candidates for optogenetic applications to switch GPCR signaling on and off with light. Additionally, we show the platform's utility for general functional GPCR assays, including assessment of G-protein activation and thermostability. We anticipate that this workflow will be a valuable resource for the GPCR research community, accelerating selection and characterization of variants with greater efficiency and reliability.

SIGNIFICANCE G-protein-coupled receptors (GPCRs) play a central role in cellular signaling and are key targets in drug discovery. However, producing functional protein remains technically challenging, often requiring extensive construct screening. Here, we present a highly efficient multiscale screening platform, based on the baculovirus expression system, that facilitates the rapid functional expression and biophysical analysis of GPCR variant libraries. This advancement significantly reduces time, effort, and cost and is broadly applicable to both basic and translational GPCR research, including drug discovery and optogenetic tool development.

INTRODUCTION

Integral membrane proteins of higher eukaryotic organisms are essential for numerous physiological processes and are implicated in many diseases. Investigating their structure-function relationships is therefore crucial for both basic biological research and pharmaceutical development (1). Among the most pharmacologically relevant membrane proteins are G-protein-coupled receptors (GPCRs), which form a large and versatile superfamily that translates a wide variety of extracellular signals into intracellular responses through the activation of heterotrimeric G-proteins (2–4).

Despite their biological significance, GPCRs are challenging targets for expression in recombinant systems and

Submitted June 13, 2025, and accepted for publication October 23, 2025.
*Correspondence: jonas.muehle@psi.ch or gebhard.schertler@psi.ch
Marielouise Griebl's present address is Molecular Biotechnology Master Program, Faculty for Engineering, Heidelberg University, Germany.
Editor: Ching-Ju Tsai.
https://doi.org/10.1016/j.bpj.2025.10.034
© 2025 The Author(s). Published by Elsevier Inc. on behalf of Biophysical Society.
This is an open access article under the CC BY license (http://creativecommons.org/licenses/by/4.0/).

functional characterization due to low yield, misfolding or aggregation, and instability in detergent micelles. Despite several successful studies, bacterial systems often fail to produce functional GPCRs, requiring more complex eukaryotic expression systems (5). Insect cells, particularly *Spodoptera frugiperda* (Sf9) and *Trichoplusia ni* (Hi5) lines, are widely used in the GPCR field due to their ability to support membrane protein folding and perform posttranslational modifications (5–9).

Nevertheless, extensive protein engineering, including stabilizing point mutations, truncations, or fusion with rigid partners, is still necessary to produce sufficient quantity and quality of these receptors to facilitate functional and structural studies (10–13). However, screening for variants with high expression or functional relevance remains a time-consuming and resource-intensive process. Currently, systematic, high-throughput workflows to assess expression levels paired with functional assays across large sets of GPCR constructs are lacking.

In this study, we present an efficient GPCR screening platform based on the baculovirus-insect cell system. Our approach enables rapid generation of high-titer baculoviruses followed by an expression screening in 96-well deep-well plates (DWPs) from 0.5-mL suspension cultures, utilizing the power of analytical fluorescence-detection size-exclusion chromatography (FSEC) (14,15). Moreover, it features the efficient assessment of stability and functionality from medium-scale (25 mL) expression cultures. The workflow includes protocols for purification and functional assessment using UV/Vis spectroscopy, G-protein activation, and thermal stability assays. Primarily driven by HPLC-based analytics, this platform enables rapid and resource-efficient identification of functionally relevant GPCR variants. Here, we used the light-sensitive GPCR jumping spider rhodopsin-1 (JSR1) as a model system to identify mutations shifting spectral properties of the receptor using the developed pipeline.

Natural light-sensitive GPCRs are pigments that mediate visual and nonvisual photoreception in animals (16). They are formed by the opsin receptor and a retinal chromophore covalently bound to the protein by a Schiff base (SB) to a conserved lysine residue. In the vast majority of animal opsins, the 11-*cis* retinal isomer acts as an inverse agonist. Upon photon absorption, the chromophore isomerizes to all-*trans* retinal, activating the receptor. In monostable opsins (e.g., vertebrate visual opsins), this activation leads to subsequent SB hydrolysis and chromophore release. In contrast, in bistable opsins (e.g., invertebrate and vertebrate nonvisual opsins), the retinal remains covalently bound and can be regenerated photochemically.

Visual pigments absorb light across a wide wavelength range, from UV ($\sim$400 nm) to red ($\sim$700 nm), depending on the electrostatic environment of the retinal binding pocket (17). A key determinant of spectral tuning is the protonation state of the SB: protonated forms absorb in the visible range, whereas deprotonated forms absorb in the UV. Protonation is stabilized by counterions, typically glutamate sidechains near the SB. Most opsins contain $E^{45.44}$ (generic GPCR numbering scheme (18) in extracellular loop 2, termed the "distal" or "ancestral" counterion. Vertebrate opsins additionally harbor a glutamic acid $E^{3.28}$ in helix 3, referred to as the "proximal" counterion.

Spectral tuning of visual pigments is critical for their application in optogenetics, a method that utilizes light-sensitive receptors to precisely trigger cellular responses in vivo and has found broad application in neuroscience (19,20). Ideally, the receptor's ground and active states should absorb at clearly separated wavelengths to allow state-specific optical control. Moreover, red-shifted absorption improves light penetration in tissues, enhancing applicability in intact organisms (21). Achieving these goals typically requires either searching for exotic visual pigments (22) or engineering of the chromophore environment to manipulate the protonation state of the SB or to stabilize specific conformations with distinct absorption maxima (23–25). These strategies can generate opsins with spectrally distinct active and inactive states, which is a desirable feature in optogenetic applications (26).

JSR1 is a bistable opsin expressed in the eyes of jumping spiders, which contributes to depth perception by signaling through the heterotrimeric Gq/11 family (27). It is an attractive model for optogenetics and biophysical and mutagenesis studies due to its exceptional thermal stability (28) and available inactive and active-state molecular structures (28,29) However, both the dark (11-*cis* retinal) and light-activated (all-*trans* retinal) states absorb at 535 nm, leading to overlapping spectra and complicating functional assays. A slight 30-nm blue shift is observed when the 9-*cis* retinal isomer is used, facilitating some distinction between states (30).

We used our methodology to systematically explore the determinants of bistability and color tuning by screening 56 JSR1 mutants in the 96-well format. After a selection step based on expression levels, we performed UV/Vis-based functional analysis on 30 variants from the 25-mL expression cultures. In this case study, we utilize an HPLC diode array detector (DAD) capable of recording full UV/Vis spectra at any time during an analytical size-exclusion chromatography run. We obtained a comprehensive data set of high-quality UV/Vis spectra in different activation states. We were able to discover five mutants of JSR1 with separated absorption spectra with potential for optogenetic control. This project demonstrates the platform's ability to efficiently screen membrane receptor variants on a small scale in a short time. Additionally, a subset of the JSR1 variants was further characterized using a G-protein activation assay and thermal stability analysis. The receptor yields from 25-mL protein expression cultures are generally sufficient to carry out multiple biochemical assays, including ligand binding studies. This highlights the

platform's potential for broader application in GPCR research and molecular pharmacology.

MATERIALS AND METHODS

Vector design

First, we constructed a vector based on the pAC8REDNK backbone (31) (kind gift of Dr. Arnaud Poterszman, Strasbourg) that harbors a P10-controlled DsRed.M1 used for assessing virus infection efficiency. We inserted a cassette for a TEV-cleavage site fused to eGFP, followed by a His_8-tag under control of a PH promoter. The vector contained NotI and BamHI restriction sites N-terminally to the TEV site for the insertion of our target proteins (Fig. S1 *A*). The TEV-eGFP-His_8 gene was synthesized and inserted by Azenta/GeneWiz (Leipzig, Germany).

JSR1 construct design

The sequence of JSR1 WT (28) was extended by a 3C protease cleavage site followed by a Twin-Strep-tag. The insertion cassette was codon optimized for Sf9 insect cells and flanked by an N-terminal NotI site and a C-terminal BamHI site. The entire gene was synthesized and inserted into the pAC8REDNK-TEV-eGFP-His_8 vector (Fig. S1 *B*) by Azenta/GeneWiz (Leipzig, Germany). Point mutants were produced by variant synthesis (Azenta/GeneWiz, Leipzig, Germany).

Insect cell cultivation in 96-well DWPs

Sf9 and Hi5 insect cells were grown in SF900II SFM (Invitrogen, California, USA) or ESF AdvanCD (Expression Systems, California, USA) insect cell medium inside autoclaved Nunc 2-mL round-well, U-bottom 96-well DWPs (Thermo Fisher, Massachusetts, USA) on a MixMate orbital shaker (Eppendorf, Hamburg, Germany) with a 3-mm shaking diameter set to 1200 rpm. The shaker was located in a TC 445S incubator (Lovibond, Dortmund, Germany) set to 27°C and humidified to approximately 70% relative humidity to avoid substantial evaporation. The plates were sealed with a sterile AreaSeal film (Sigma-Aldrich, Buchs, Switzerland). Cell fitness parameters were assessed on the CellDrop BF cell counter (Denovix, Delaware, USA) by mixing 10 μL of cell suspension with 10 μL of 0.4% Trypan Blue solution (Gibco).

Virus production in 96-well DWPs

500 ng plasmid DNA was mixed with 100 ng linearized Bac10:KO1629 viral DNA in 25 μL SF900II SFM medium inside the well of a 96-well DWP. A master mix of 1.2 μL Cellfectin II (Invitrogen) in 25 μL SF900II SFM per transfection reaction was prepared, added to the plasmid/viral DNA mix, and incubated for 20–30 min at room temperature. Afterward, 8.0×10^5 live Sf9 cells in 450 μL medium were pipetted on top of each transfection mix. The plate was sealed with an AreaSeal film (Sigma-Aldrich, Buchs, Switzerland) and incubated for 5 h at 27°C and 1200 rpm (3-mm orbital). Then, 500 μL SF900II SFM medium supplemented with 2% Penicillin/Streptomycin/Amphotericin B (PAN Biotech, Aidenbach, Germany) was added to each well to dilute the Cellfectin II, and the plate was sealed and incubated again as mentioned above. After 4 days of incubation, V_0 generation virus was harvested by centrifugation at 800 × *g* and 20°C, supplemented with 1% Penicillin/Streptomycin/Amphotericin B and 10% FCS (Sigma-Aldrich, Buchs, Switzerland), and stored in the dark at 4°C.

Viruses were amplified twice by infecting 1.0 mL of fresh Sf9 cultures at 2.0×10^6 live cells/mL in SF900II SFM medium with 1% V_0 or V_1 generation viruses, respectively. High-titer V_1 or V_2 generation viruses were harvested 72 h after infection by centrifugation as described above. However, the concentration of FCS was reduced from 10% to 1%.

FSEC expression screening

500 μL of Sf9 or Hi5 insect cells (Invitrogen) in ESF AdvanCD medium (Expression Systems, California, USA) at a density of 3.0×10^6 live cells/mL was infected with 5% (v/v) V_2 generation virus stocks inside a 96-well DWP. The plate was sealed with an AreaSeal film and incubated for 48 h at 27°C and 1200 rpm (3-mm orbital). Cells were harvested by centrifugation at 1500 × *g* and 20°C, and the supernatant was homogenously removed with a VialFlow 96-channel pipette (Integra Biosciences, Zizers, Switzerland). Pellets were frozen and stored inside the 96-well DWP until further use at −20°C.

For the FSEC screening, cells were thawed and solubilized in 200 μL solubilization buffer (20 mM HEPES (pH 6.5), 300 mM NaCl, 1 mM $MgCl_2$, 100 μM TCEP, DNAse I, Roche EDTA-free cOmplete protease inhibitor (1 pill/50 mL), 1% LMNG, and 0.1% CHS) for 90-min shaking at 4°C and 2000 rpm (3-mm orbital). Insoluble material was removed by ultracentrifugation for 40 min at 4°C and 200,000 × *g* using a Type 42.2 Ti rotor (Beckman Coulter, Nyon, Switzerland). To avoid pipetting errors, we used a custom-made ultracentrifuge tube holder in a 96-well design made of aluminum (Fig. S2). The clarified crude solubilized fractions were transferred into a V-bottom-shaped 96-well plate (Greiner Bio-One, St. Gallen, Switzerland) for injection into a 1260 Infinity II HPLC system (Agilent, California, USA), which was preequilibrated in FSEC buffer (20 mM HEPES (pH 6.5), 300 mM NaCl, 100 μM TCEP, 0.02% LMNG, and 0.002% CHS). 10 μL of each sample was injected onto a Zenix-C SEC300 4.6 × 150 mm column (SEPAX Technologies, Delaware, USA) at 20°C and a flow rate of 0.5 mL/min. The GFP fluorescence was measured on a 1260 Infinity II Fluorescence Detector Spectra (cat. number G7121B) (Agilent, California, USA) using 488-nm excitation wavelength, 512-nm emission wavelength, and a GAIN of 14.

The FSEC traces were baseline-corrected and integrated using the OpenLab CDS v2.8 software (Agilent, California, USA). The software-internal "peak explorer" function was used to represent the chromatograms as bubble plots for a simplified analysis.

Small-scale expression and purification

25 mL of Hi5 insect cells (Invitrogen) in ESF AdvanCD medium (Expression Systems, California, USA) at a density of 3.0×10^6 live cells/mL were infected with 1% (v/v) V_2 generation virus stocks inside TubeSpin Bioreactors 50 (TPP, Trasadingen, Switzerland). Proteins were expressed for 48 h at 27°C and 250 rpm (25-mm orbital shaker). Cells were harvested by centrifugation at 1500 × *g* and 20°C, and the supernatant was discarded. Pellets were frozen in liquid nitrogen and stored at −80°C.

All following steps were carried out at 4°C or on ice unless otherwise stated. Frozen cell pellets were thawed in 20 mL resuspension buffer (20 mM HEPES (pH 6.5), 300 mM NaCl, 1 mM $MgCl_2$, 100 μM TCEP, DNAse I, Roche EDTA-free cOmplete protease inhibitor (1 pill/50 mL)), releasing soluble proteins such as DsRed.M1. The insoluble fraction was pelleted by ultracentrifugation for 40 min at 200,000 × *g* and the supernatant discarded. The pellet was then mechanically disrupted in 20 mL resuspension buffer using an Ultra-Turrax (IKA, Staufen, Germany) for 1 min at 11,000 rpm to lyse the cells. Membranes were collected by ultracentrifugation under the same conditions, and the supernatant was discarded. The membrane pellet was homogenized in 2.8 mL lysis buffer using an Ultra-Turrax as described above. The resulting suspension was frozen in liquid nitrogen and stored at −80°C until further use.

All following steps were additionally carried out under dim red-light conditions. Thawed membrane suspensions were incubated for 60 min with 50 μM 9-*cis* retinal to reconstitute the receptors before solubilization for 90 min in 1% LMNG and 0.1% CHS. The lysate was clarified by

ultracentrifugation for 40 min at 200,000 × g and the pellet discarded. The supernatant was incubated with 50 μL homemade anti-GFP-Nanobody (32) resin or Streptactin XT resin (IBA Lifesciences, Göttingen, Germany), respectively, for 60–90 min. The resin was collected by centrifugation at 1000 × g for 3 min, and the supernatant was carefully removed. The resin was resuspended in 500 μL wash buffer (20 mM HEPES (pH 6.5), 300 mM NaCl, 100 μM TCEP, 0.05% LMNG, and 0.005% CHS) and transferred to Proteus 1-Step Batch Mini Spin columns (Protein Ark, Rotherham, UK). The resin was washed three times with 500 μL wash buffer for 1 min at 12,000 × g. The receptors were proteolytically eluted from the resin in 100 μL elution buffer (wash buffer + 0.05 mg/mL homemade HRV 3C protease) for 60 min.

UV/Vis analysis

UV/Vis spectra were recorded on an HPLC Infinity II DAD WR (cat. number G7115A) (Agilent, California, USA) equipped with a 10-mm flow cell during an analytical SEC run. 10–20 μL of the purified receptors was injected at 20°C and a flow rate of 0.5 mL/min onto a Zenix-C SEC300 4.6 × 150 mm column (SEPAX Technologies, Delaware, USA), which was preequilibrated in SEC buffer (20 mM HEPES (pH 6.5), 300 mM NaCl, 100 μM TCEP, 0.02% LMNG, and 0.002% CHS). Chromatograms were recorded at 280 nm and up to seven additional variable wavelengths. Simultaneously, UV/Vis spectra were recorded from 250 nm to 750 nm with 1-nm resolution at any time of the SEC run. The UV/Vis spectra were extracted from the chromatograms at the peak position of the receptor peak within the Agilent OpenLab CDS v2.8 software package and normalized to the global absorption maximum at 280 nm.

To obtain spectra of different activation states, the samples were illuminated for 1 min with 100% intensity using the Optowell 24 (Optobiolabs, Freiburg, Germany) at 365 nm (0.49 mW/cm^2), 460 nm (2.74 mW/cm^2), or 520 nm (1 mW/cm^2), depending on the chromophore absorption maximum of each variant. Every recorded spectrum required an individual SEC run as described above.

For the performance comparison of the DAD to our standard spectrophotometer, UV/Vis spectra were recorded as described elsewhere (33).

G-protein activation assay

The GTPase-Glo assay (Promega, Madison, USA) was performed as described elsewhere (34). However, we used the prenylated Gq heterotrimer instead of a soluble version of heterotrimeric G11. Briefly, 2.5 μL of protein solution containing 2 μM heterotrimeric Gq, 0.2 μM dark-state or light-activated JSR1 variants was mixed with 2.5 μL of 2 μM GTP solution. The reaction was conducted for 180 min while shaking at 500 rpm. Subsequently, 5 μL of GTP-Glo reagent was added. After 30 min, 10 μL of detection reagent was added. Luminescence was measured in a PheraStar FSX (BMG Labtech, Ortenberg, Germany) plate reader. Each condition was prepared with $n = 6$.

Thermal stabilization assay

JSR1 mutants, purified from a 25-mL expression culture, were diluted to a final concentration of 0.5 μM in 300 μL of SEC buffer (20 mM HEPES (pH 6.5), 300 mM NaCl, 100 μM TCEP, 0.02% LMNG, and 0.002% CHS). Half of each sample was illuminated for 1 min with 100% intensity using the Optowell 24 (Optobiolabs, Freiburg, Germany) at 460 nm (2.74 mW/cm^2) or 520 nm (1 mW/cm^2), depending on the chromophore absorption maximum of each variant. The samples were equally distributed over seven aliquots. Each aliquot was incubated for 5 min at one of the following temperatures: 4°C, 30°C, 40°C, 50°C, 60°C, 70°C, or 80°C using a Dri-Block DB-2D Digital block heater (Techne). Subsequently, the samples were cooled on ice and centrifuged for 2 min. Then, the samples were injected onto a Zenix-C SEC300 column as described above. In addition to the UV_{280nm}, UV/Vis spectra from 250 to 750 nm were recorded at 1-nm resolution. The peak maximum of each chromophore peak was plotted against the corresponding incubation temperature. To determine the melting point Tm, the data were fitted with a Boltzmann sigmoidal function:

$$y = \frac{A1 - A2}{1 + \exp\left(\frac{x - Tm}{dT}\right)} + A2$$

Here, y = observed peak maximum; x = temperature; A1 = upper asymptote; A2 = lower asymptote; Tm = melting temperature; dT = slope factor.

RESULTS

Efficient GPCR variant selection enabled by a multiscale screening workflow

Our high-throughput screening platform (Fig. 1) streamlines the assessment of expression levels and functional properties of a large number of protein variants in parallel. The workflow enables systematic and rapid protein characterization, allowing for a data-driven decision regarding the upscaling of the most promising and interesting targets for further large-scale production, thereby enhancing the likelihood of solving research questions more quickly.

After an extensive period of testing, our workflow (shown in Fig. 1) involves high-titer baculovirus production from suspension cultures in 96-well DWPs, followed by FSEC (14,15) expression screening on an HPLC system equipped with an autoinjector and fluorescence detector. This process allows for the exclusion of nonexpressing or poorly expressing proteins. The selected variants will be expressed in 25-mL expression cultures using highly suitable TPP TubeSpin Bioreactors 50 (Falcon tube size). The expressed protein variants will be purified in a small-scale affinity purification. Subsequently, they can be characterized functionally using UV/Vis spectroscopy, thermostability, and/or functional assays, providing valuable insights for selecting the most promising research targets. Our laboratory works on membrane proteins, specifically on light-sensitive GPCRs, which are challenging targets. We therefore exemplify our procedures on bistable visual pigments used for protein engineering. The workflow shown in Fig. 1 enabled the efficient generation and characterization of 56 JSR1 constructs—far more rapidly than conventional sequential approaches would allow.

Miniaturized transfection and virus production protocols for suspension cultures

Conventionally, transfection for virus production is executed in adherent cultures. To enable high-throughput workflows and facilitate the use of multichannel pipettes, we aimed to develop a protocol in which all steps, including transfection and virus amplification, are performed in suspension. We established reproducible

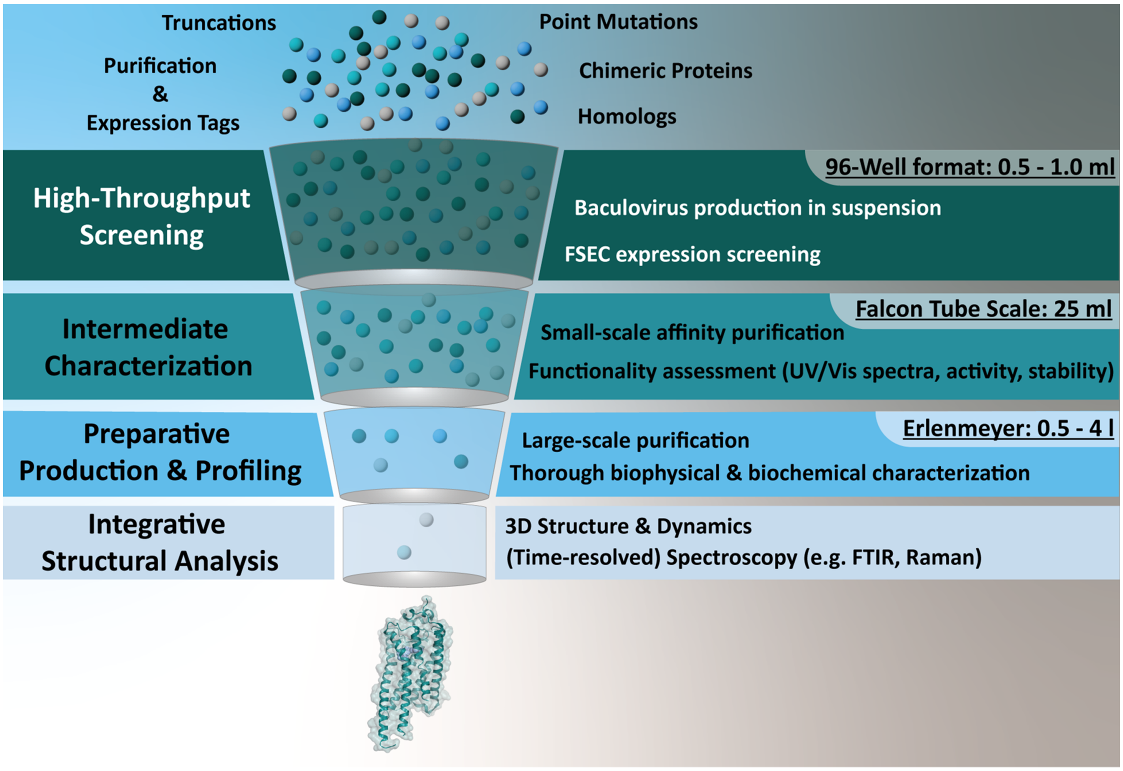

FIGURE 1 Schematic representation of the modular multiscale screening platform. The workflow enables systematic and parallel small-scale baculovirus production and FSEC-based expression screening for a large number of protein constructs, including protein homologs, truncated proteins, protein chimeras, or other modified proteins such as point-mutated residues or differently tagged proteins. Based on this first assessment of expression levels, the most promising candidates can be upscaled in a second step to a 25-mL culture volume. This upscaling allows the simultaneous production of dozens of pellets, yielding enough material for a small-scale purification and a first investigation of the functional properties of the different protein variants. The types of assays that can be performed at this stage include the analysis of purity and homogeneity along with the UV/Vis absorption properties (HPLC-SEC with DAD), thermal stability (e.g., HPLC-based/FSEC-TS, CPM, or NanoDSF), functionality (e.g., G-protein activation), and protein-protein/-ligand interactions. Depending on the expression levels and yields of different targets, several of these assays can be performed from a single small-scale purification. The results of the first two screening phases should provide a solid foundation for the data-based selection of the most promising and interesting mutants for upscaling. Several variants can be upscaled in parallel for expression and purification. The purified proteins can be used for a more thorough biophysical and biochemical characterization and for the desired type of analysis, which might include the elucidation of the 3D structure and the structural dynamics, as well as a variety of spectroscopic measurements. This study focuses on the "high-throughput screening" and the "intermediate characterization" stages.

suspension culturing conditions for Sf9 and Hi5 insect cells in 96-well DWPs. To optimize mixing and aeration of the cultures, we calculated the ideal shaking frequency by applying the knowledge from microbial growth inside multiwell plates (35,36). We found that optimal growth was achieved at 1200 rpm on a 3-mm orbital shaker in 2-mL DWPs with round wells and U-shaped bottoms. Below this shaking frequency, sedimentation of Hi5 cells hindered efficient cell growth. Additionally, baculovirus-infected, swollen Sf9 cells exhibited a tendency to sediment, causing irreproducible results.

In the next step, we developed transfection conditions in suspension inside 96-well DWPs based on the homologous recombination technology (37) using red and green fluorescent proteins as indicators for transfection efficiency (31) (Fig. S1 *A*). Several different transfection reagents were tested. We observed the most efficient transfection with Cellfectin II. We were able to reduce the amount of Cellfectin II to 1.2 μL per reaction without a noticeable loss of transfection efficiency compared with the usual 8 μL commonly employed in conventional protocols for adherent cultures (Fig. S3 *A–D*). Furthermore, we compared the strength of virus stocks produced with our method in DWPs with those produced using conventional protocols, utilizing an endpoint dilution assay, with which we determined the tissue culture infectious dose 50% per ml value (38). Both virus amplification methods yielded similarly strong V_1 virus titers with tissue culture infectious dose 50% per ml values exceeding 1×10^8 (Fig. S3 *E*).

Case study: Mutations in the JSR1 retinal binding site

To test the applicability of the transfection and virus amplification protocols, we started a case study on JSR1, aiming to shed light on the determinants of bistability in visual pigments as well as color tuning for optogenetic

applications. Therefore, we inserted the JSR1 gene fused to a cleavable Twin-Strep-tag into the pAC8RED plasmid linking the gene to the eGFP cassette (Fig. S1 *B*). We produced 56 JSR1 variants, mainly focusing on the mutations near the SB in the receptor's retinal binding site (Fig. 2 *A*). Namely, we mutated the residues $Y126^{3.28}$ and $S199^{45.49}$. Interestingly, $S199^{45.49}$ has been shown to be relevant for the protonation state of the ground state of JSR1 (26,29). The $S199F^{45.49}$ mutation induces a deprotonation of the ground state, leading to separated absorption maxima of ground and active states. Unlike JSR1 WT, this mutation enables the possibility to selectively switch the receptor on and off using different illumination wavelengths. $Y126^{3.28}$ is in a position in the retinal binding pocket typically occupied by a glutamate in monostable opsins such as human rhodopsin. This glutamate has been suggested to play a regulatory role in the SB hydrolysis preceding retinal release (39). We hypothesized that this residue might be a key determinant for bistability and an interesting target for color-tuning mutations. However, preliminary experiments revealed inconsistent expression and retinal incorporation for a few rationally designed mutants. To systematically address this uncertainty, we generated a complete substitution library by introducing all 20 amino acids at each position ($Y126^{3.28}$ and $S199^{45.49}$). This approach aimed to identify well-expressing variants suitable for reliable functional and spectroscopic analysis.

Virus production in a 96-well suspension format

We successfully produced high-titer V_2 generation baculovirus stocks for all 56 JSR1 variants using the established protocols in a short time (Fig. 2 *B*). Initial transfections generated V_0 baculovirus stocks, which were used to produce V_1 viruses. Although virus titration assays (Fig. S3 *E*) indicated consistently high and reproducible titers, we observed some variability in infection levels across wells (Fig. 2 *B*, middle panel). To ensure consistent infection levels, we used the V_1 generation viruses to generate final V_2 generation baculoviruses (Fig. 2 *B*, right panel).

The red infection marker (Fig. S1 *A*) helped to identify transfected or infected cultures by visual inspection (Fig. 2 *B*). We noticed a progressive increase in red hue during the virus stock amplifications. In the V_2 amplification, the red hue reached its maximum and was similar across all wells. All negative controls (wells G8–11) remained beige, indicative of uninfected cells (Fig. 2 *B*). Randomly selected wells across the plate were additionally inspected by fluorescence microscopy confirming the successful expression of the infection marker as well as the target proteins by employing the green fluorescence protein expression marker (Fig. S1 *B*). We noticed a poor infection efficiency in column 4 after V_1 amplification, likely caused by a pipetting error with the multichannel pipette used to transfer V_0 virus to the new plate. However, after V2

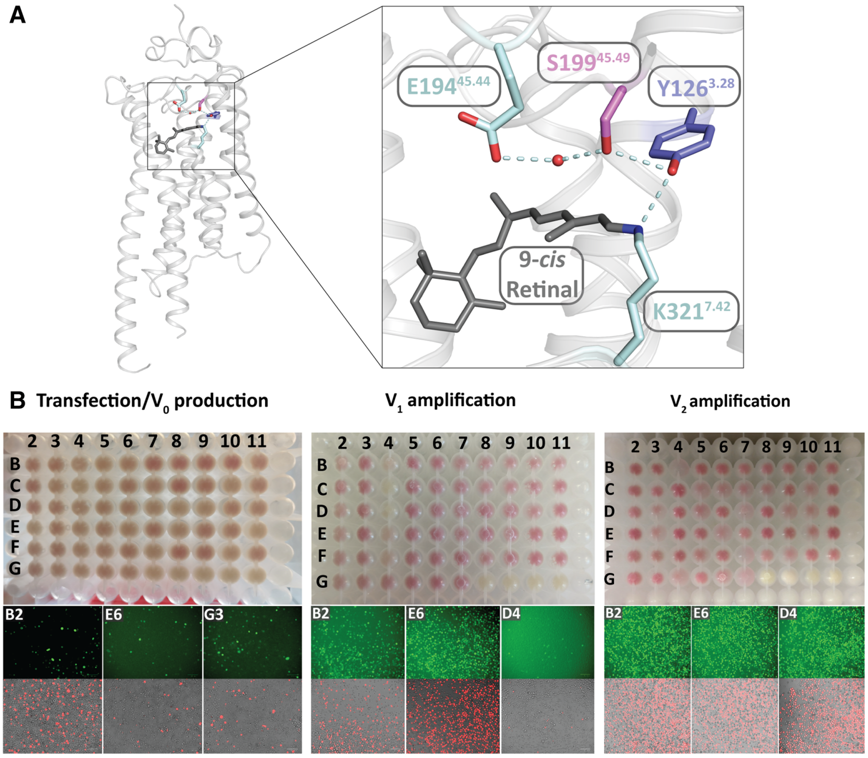

FIGURE 2 Virus production for mutagenesis case study. (*A*) Structure of JSR1 (PBD: 6I9K) depicted as gray cartoon and close-up view of the retinal binding pocket showing residues $K321^{7.42}$, $E194^{45.44}$, $Y126^{3.28}$, and $S199^{45.49}$, and 9-*cis* retinal as sticks. A water molecule is depicted as a small red sphere. Cyan dotted lines indicate polar interactions of a hydrogen bond network. (*B*) Successful virus production steps for all 56 JSR1 viruses indicated by the expression of the red infection marker (DsRed.M1). In addition to the $Y126X^{3.28}$ and $S199X^{45.49}$ mutations, we performed an alanine scanning mutagenesis of all cysteines in JSR1 to address other research questions, which will not be discussed in this manuscript. However, these mutants were part of the virus production procedure and are shown in the images above. Column "4" shows minor signs of infection after the V_1 amplification (middle panel), likely due to a pipetting mistake with the multichannel pipette. However, all V_2 generation viruses (right panel) seem to be equally potent, as indicated by the fluorescence microscopy images of randomly selected wells across the plate. Initially, we did not select well D4 for fluorescence microscopy. Therefore, well G3 is shown instead for the V_0 production. However, we replaced it with D4 for the following virus amplification steps to monitor infection levels in the aforementioned poorly infected column.

amplification, all viruses on the plate appeared to induce infection.

Efficient screening for functional receptor expression

For the FSEC expression test, we used the 56 V_2 virus stocks to infect 500 μL of fresh Hi5 cells in 96-well DWPs with a saturating amount of virus (5% (v/v)), guaranteeing primary infections for all cells to maximize the protein expression. If the number of mutants allows it, it is advisable to screen several virus concentrations at this stage to account for possible differences in the virus titers. The cell pellets yielded from this expression test are solubilized in detergent and subjected to ultracentrifugation before injection into the HPLC system (Fig. 3 *A*, left panel). Since all constructs are GFP-fusion proteins (Fig. S1 *B*), we can selectively detect our proteins in the crude solubilized fraction utilizing the fluorescence detector. The FSEC screening typically provides information on the aggregation as well as the expression levels, the amount of “functional” protein, and its oligomerization state (Fig. 3 *A*, middle panel). All steps were performed in the JSR1 apo state under light conditions. However,

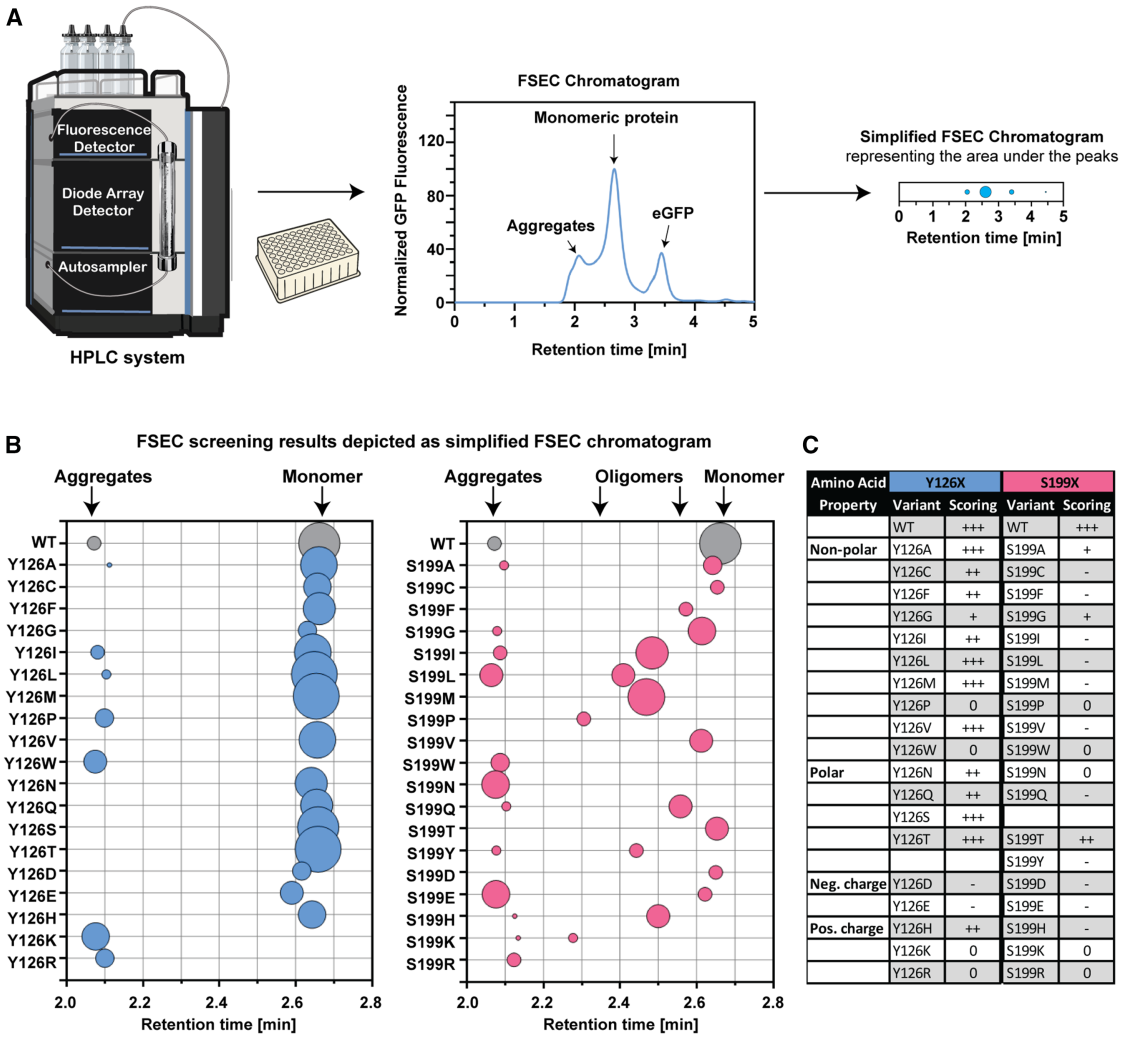

C

Amino Acid Property	Y126X Variant	Y126X Scoring	S199X Variant	S199X Scoring
	WT	+++	WT	+++
Non-polar	Y126A	+++	S199A	+
	Y126C	++	S199C	-
	Y126F	++	S199F	-
	Y126G	+	S199G	+
	Y126I	++	S199I	-
	Y126L	+++	S199L	-
	Y126M	+++	S199M	-
	Y126P	0	S199P	0
	Y126V	+++	S199V	-
	Y126W	0	S199W	0
Polar	Y126N	++	S199N	0
	Y126Q	++	S199Q	-
	Y126S	+++		
	Y126T	+++	S199T	++
			S199Y	-
Neg. charge	Y126D	-	S199D	-
	Y126E	-	S199E	-
Pos. charge	Y126H	++	S199H	-
	Y126K	0	S199K	0
	Y126R	0	S199R	0

FIGURE 3 Analytical FSEC expression screening for mutagenesis case study. (*A*) HPLC-based FSEC expression test and data simplification for optimized analysis, enabling the quick observation of trends and preselection of mutants from large data sets. Peaks in the chromatograms are integrated, and each peak is represented as a bubble at the corresponding retention time. The size of the bubbles correlates with the area under the curve. (*B*) Simplified FSEC chromatograms represented as a bubble plot of all JSR1 Y126X and S199X variants produced in Hi5 insect cells. Gray bubbles represent the expression level of the JSR1 WT reference, whereas the Y126X mutants are shown in blue and the S199X mutants in magenta. The different variants are stacked on the y-axis of the plot. (*C*) Scoring of the JSR1 mutants’ expression levels based on the simplified chromatograms, but also on the analysis of the individual FSEC traces. “+++” to “+” scores represent the expression levels based on the area under the monomeric protein peak: “+++” = similar to JSR1 WT, “++” = approximately 80%–50% of JSR1 WT, “+” = approximately 50%–25% of JSR1 WT. The “–” score might either describe poorly expressing monomeric variants with expression levels <25% of JSR1 WT or oligomerizing proteins regardless of their expression levels. The “0” score is given to none-expressing mutants.

less stable receptors might first require reconstitution with retinal or a stabilizing ligand.

This analysis yielded a large number of chromatograms. Typically, each chromatogram contains a peak for the aggregates, the monomeric receptor, and free eGFP (Fig. 3 *A*, middle panel). To enhance the analysis of all chromatograms, we simplified each chromatogram to a bubble plot by integrating each peak and plotting a bubble at the corresponding retention time with a bubble size relative to the area under the peak (Fig. 3 *A*, right panel, and *B*). The simplified plot provides an instant overview of trends in large data sets and the required information for preselecting the best-expressing, monomeric variants (Fig. 3 *B*). However, it does not replace the analysis of the individual FSEC chromatograms by an experienced scientist. An overview of the expression result is provided in Fig. 3 *C*. The data for the monomeric receptor peak show higher expression levels for the mutations at position Y126$^{3.28}$ compared with the mutations at residue S199$^{45.49}$. This finding indicates a pronounced functional role of S199$^{45.49}$. Moreover, we identified a subset of amino acids, namely arginine, tryptophan, proline, and lysine, that were not tolerated in either of the two positions (Fig. 3 *C*). Their bulky side chains and/or disruptive chemical properties likely interfere with the local protein architecture within the tight retinal binding pocket, leading to protein instability and misfolding.

HPLC-based characterization of the UV/Vis absorption properties from 25-mL expression cultures

The FSEC expression test provides the first stage of target selection for the 25-mL-scale expression. Typically, the best-expressing constructs are further investigated. In our case, to learn about the limits of the downstream analysis, we proceeded with all JSR1 variants that showed expression (Fig. 3 *C*). 25 mL of Hi5 insect cells in TPP TubeSpin Bioreactors 50 were infected with V_2 virus stocks from the 96-well DWP. Our shaker setup allowed the parallel expression of up to 48 constructs. A small-scale affinity purification workflow with 9-*cis* retinal-reconstituted JSR1 was established that yielded 100 μL of highly pure JSR1 variants with typical concentrations between 1 and 10 μM (Fig. S4). Essential in this workflow is the elution from the affinity resin by HRV 3C protease cleavage for removal of the fluorophore, which would strongly interfere with the spectral assessment of the JSR1 mutants, obtaining an expression-tag-free protein.

UV/Vis spectra of the mutants were recorded on an HPLC during an analytical size-exclusion chromatography run. Crucial in this system was a highly sensitive UV/Vis DAD, which enables recording full UV/Vis spectra throughout the SEC run (Fig. 4 *A*–*C*). The chosen setup uniquely enabled simultaneous assessment of the purity and homogeneity of JSR1 variants (Fig. 4 *B*) together with high-quality spectral absorption measurements (Fig. 4 *C*). By extracting spectra specifically at the peak maximum of the SEC run (indicated with an asterisk in Fig. 4 *B*), representing the purest subpopulation of each mutant, we obtained exceptionally accurate and detailed spectral information, combining two analyses that are typically performed separately. Each run required only 10 μL at a concentration of $\geq$0.25 μM of the affinity chromatography-purified protein solution. Hence, 100 ng of purified JSR1 was sufficient to record UV/Vis spectra of high quality. We compared the data quality with our standard benchtop spectrophotometer (Fig. S5) and observed an improved signal/noise ratio of the DAD detector, particularly in the region from 300 to 400 nm (Fig. S5 *B*). Moreover, we found a drastically enhanced chromophore absorption signal upon spectrum extraction at the JSR1 elution peak (Fig. S5 *C*).

The established small-scale workflow (Fig. S4) enables the purification and analysis of 20 protein mutants per week with a moderate workload. However, this multiplexing poses a certain degree of complexity on the data analysis, as it produces a large number of spectra. We found that plotting the spectra as a heat map (Fig. 4 *C* and *D*) allowed for an intuitive comparison of the absorption spectra. It facilitated displaying the entire set of JSR1 mutants in their 9-*cis* retinal-bound ground state (“DARK”) as well as their spectral absorption features after a first and second illumination step in a single plot (Fig. 4 *D*). The data demonstrate that all expressible JSR1 variants could be purified and analyzed regardless of their expression levels from the FSEC screening. Even low-expressing mutants yielded enough material to record spectra of several activation states. We observed that all mutants incorporated 9-*cis* retinal successfully. Moreover, we found that the λ_{max} of all mutants changes upon a first illumination step, indicating a functional fold of the proteins. In addition, this large data set allowed us to gain valuable insights into color-tuning mechanisms and the determinants of bistability. Specifically, all mutants retained their bistability, including the Y126E$^{3.28}$ mutant.

With respect to color tuning, we found that all Y126$^{3.28}$ mutants exhibited WT-like behavior, although they were slightly blue-shifted to varying extents (Figs. 4 *D* and S6). The most similar mutant to the wild-type was Y126F$^{3.28}$, which exhibited only a minor blue shift in both the ground and excited state. Interestingly, although Y126H$^{3.28}$ showed the largest blue shift in the 9-*cis* ground state, it deviated least from the WT in the activated state, leading to an enlarged spectral separation. However, like all other Y126X$^{3.28}$ mutants, a second pulse of illumination with green light did not restore λ_{max} of the 9-*cis* ground state.

Intriguingly, the impact on the protonation state as seen from the altered spectral properties was significantly higher for the S199X$^{45.49}$ mutants. This finding aligns well with the reduced expression levels from the FSEC screening, which suggested a pronounced functional relevance of S199$^{45.49}$

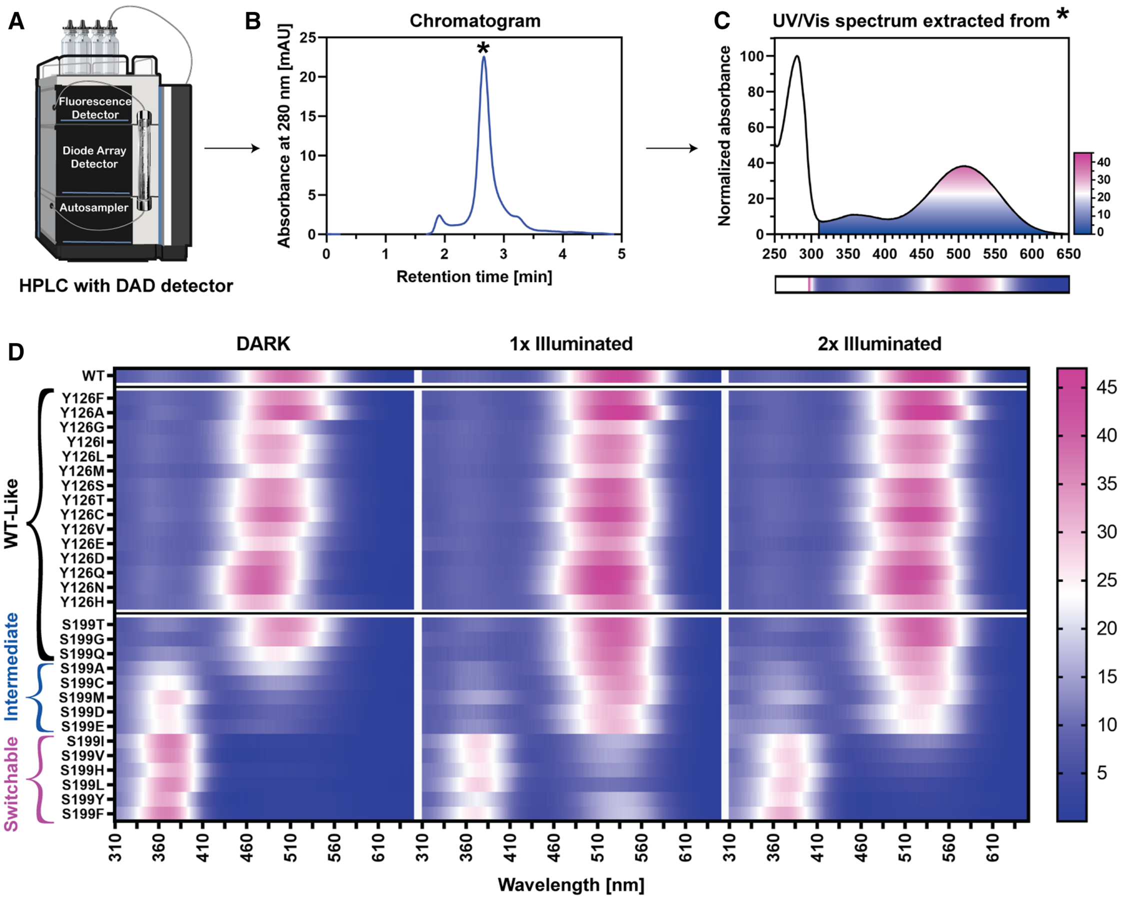

FIGURE 4 Analysis of the UV/Vis spectral properties of all expressible JSR1 variants. (*A*) HPCL equipped with a diode array detector (DAD) is the core instrument of the analysis. The detector enables recording and extraction of full UV/Vis spectra at any time of the run. (*B*) Chromatogram of a small-scale purified JSR1 variant showing a highly clean protein with very small amounts of aggregates after a single affinity-purification step. The star above the peak apex shows the position at which the UV/Vis spectra were extracted. (*C*) UV/Vis spectrum of a JSR1 variant normalized to the UV280 signal extracted from the peak maximum in the chromatogram. A region of interest from 310 nm to 650 nm was defined. Within this region, the signal intensity was converted into a color by a blue-white-magenta color gradient reaching from 0 to 45% in relationship to the UV280 signal as indicated on the spectrum. This allowed for a simplified heat map illustration shown below the spectrum. (*D*) Overview of all UV/Vis spectra (collected at the center of the monomeric peak) of the JSR1 mutants represented as heat maps. Each bar corresponds to a full UV/Vis spectrum. For each variant, a spectrum in its ground state bound to 9-*cis* retinal was recorded. Additionally, spectra from two rounds of illumination with wavelengths near λ_{max} are shown. The proteins could be classified into three categories: 1) "wild-type-like"—the proteins had a λ_{max} in the blue/green (470–500 nm) region in the 9-*cis* ground state. Upon illumination, λ_{max} was red-shifted to the green range (520–535 nm) and remained largely unchanged upon a second illumination. 2) "Intermediate"–the 9-*cis* ground state was shifted partially or fully to the UV range (370 nm), indicating deprotonation of the SB. A first illumination with UV light triggered the protonation of the active state, and a shift of λ_{max} was to the green range (520–535 nm). A second illumination with 520-nm light did not return the signal back to the UV range. The difference might arise from differences between 9-*cis* (start) and 11-*cis* (after back illumination) retinal. 3) "Switchable"—entirely deprotonated SB in the ground state. After illumination with UV light, a partial protonation was observed. The second illumination with green light induced a full deprotonation.

compared with Y126$^{3.28}$. A few amino acids, specifically threonine, glycine, and glutamine, displayed a wild-type-like behavior as described for the Y126X$^{3.28}$ mutants. All other variants showed a strong tendency for deprotonation of the SB and a spectral shift to the UV range. Interestingly, the data indicated two subpopulations within this set of mutations. The first one is composed of the amino acids alanine, cysteine, methionine, aspartate, and glutamate. All of them exhibited two absorption maxima: the main peak around 360 nm and a second one near 500 nm in the 9-*cis* retinal-bound ground state with varying intensity ratios. Upon activation with UV light, λ_{max} transitioned predominantly to the green range around 525 nm. A second illumination with 520-nm light induced only a mild shift back to the UV range, whereas the main peak remained unchanged. We speculate that the strong deprotonation exclusively occurs for 9-*cis* retinal but not for 11-*cis* retinal, which is the expected photoproduct after the second light pulse. Potentially, the ability to separate the absorption maxima of the inactive and active states for those mutants could be fine-tuned by modifying the pH (26). The second subpopulation is composed of the amino acids isoleucine, valine, histidine, leucine, tyrosine, and phenylalanine. These mutations cause a full deprotonation in the 9-*cis* retinal-bound

ground state and become partially protonated upon light activation. A second light pulse switches the λ_{max} back to the UV range as described for the S199F$^{45.49}$ mutation (26). We did not observe a clear trend in the influence of the spectral features based on the physical properties of the amino acids.

General GPCR assays for functional characterizations from 25-mL expression cultures

Orthogonal biophysical or biochemical insights, like thermal stability or G-protein activation, can significantly assist in the target selection step for upscaling (Fig. 1). Moreover, such data can be valuable for non-opsin-GPCRs if UV/Vis spectra cannot be used for a functional description of a protein. Hence, we explored the possibility of performing other assays for a functional characterization using the material yielded from 25-mL expression cultures.

Accurate estimation of protein yields was essential to ensure defined stoichiometries in downstream assays. To achieve this, we generated a calibration curve on the analytical SEC using a purified and quantified JSR1 WT stock (Fig. 5 *A*). Based on this, we purified JSR1 WT and five Y126X mutants in parallel from 25-mL cultures and quantified their yields using the established calibration curve. The yields varied from approximately 3 to 10 μM in 100 μL, corresponding to a total amount of 13–41 ng JSR1 (Fig. 5 *B*). From these amounts, we recorded the UV/Vis spectra in the three functional states as described above. The residual protein was used to perform the GTPase-Glo assay (Promega) and SEC-based thermal stability assay (40). The GTPase-Glo assay required 40 μL of JSR1 protein solution at 0.4 μM to record data in the ground state and the activated state. The data show that in absence of a receptor, the heterotrimeric Gq protein exhibits basal GTP depletion activity of approximately 32%, which is slightly reduced in presence of the JSR1 variants in the ground state. Upon light activation, all mutants specifically induce GTP depletion by the Gq protein, demonstrating the functionality of all variants (Fig. 5 *C*). To reduce the receptor consumption, we used a 10-fold molar excess of G-protein over JSR1. Depending on the yields of the target protein, the assay conditions might require optimization, such as lowering the G-protein concentration and the molar excess of G-protein to increase

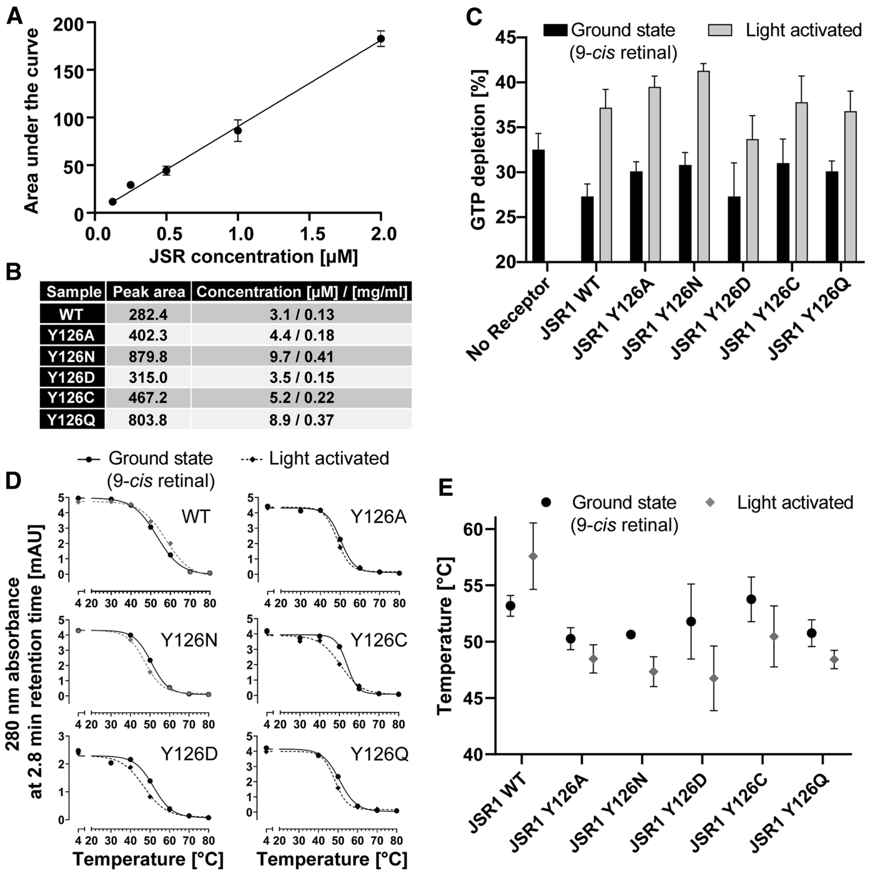

B

Sample	Peak area	Concentration [μM] / [mg/ml]
WT	282.4	3.1 / 0.13
Y126A	402.3	4.4 / 0.18
Y126N	879.8	9.7 / 0.41
Y126D	315.0	3.5 / 0.15
Y126C	467.2	5.2 / 0.22
Y126Q	803.8	8.9 / 0.37

FIGURE 5 Biochemical and biophysical characterization of GPCRs purified from 25-mL expression cultures. (*A*) Analytical size-exclusion chromatography column calibration curve. A JSR1 WT standard with a known concentration was serially diluted and injected onto a SEPAX Zenix-C SEC300 4.6 × 150 mm column in duplicates. The JSR1 peaks of the chromatogram were integrated, the area under the curve plotted against the concentration, and a calibration curve extrapolated by linear regression (*solid line*). Dots and error bars represent the mean ± SEM. (*B*) Calculated concentrations of JSR1 variants purified from 25-mL expression cultures and eluted in 100 μL elution buffer. Due to technical problems during the affinity chromatography binding step, a significant fraction (>50%) of JSR1 WT and Y126A$^{3.28}$ was lost in the flow-through, explaining the unexpected low yields for otherwise well-expressing variants. (*C*) GTPase-Glo assay measuring specific receptor-induced activation of a heterotrimeric Gq. Black bars show basal Gq activity in the absence ("no receptor") or presence of JSR1 variants in the 9-*cis* retinal-bound ground state. Gray bars show the increased GTP depletion activity of Gq after light activation of the JSR1 variants. Data represent mean ± SEM values of six replicates. (*D*) HPLC-derived melting curves of different JSR1 mutants obtained from the decay of the UV_{280nm} absorption at increasing temperatures (see Fig. S7). The data represent the absorption peak height at the monomeric peak position at the corresponding temperature. Values from the ground state proteins are shown as black dots, whereas data after light activation are shown as gray rhombuses. The data were fitted by a Boltzmann sigmoidal equation. Fitted curves are shown as black solid (ground state) and gray dashed (active state) lines. (*E*) Melting temperatures calculated from the melting curves in (*D*). The melting points are shown as black dots (ground state) and gray rhombuses (light-activated state). Error bars indicate the upper and lower limits of the 95% confidence interval of the fit.

the assay window and help identify differences between the mutants.

To conduct the thermal stability assay, we prepared 300 μL of protein solution at 0.5 μM. Only mutant Y126D$^{3.28}$ had insufficient amounts to achieve the required volume at 0.5 μM. The protein was subsequently diluted to 0.3 μM. 15-μL aliquots of each protein were incubated at 4°C and at increasing temperatures starting from 30°C with 10°C increments until 80°C. Protein thermal degradation was monitored by analytical SEC through recording $UV_{280\ nm}$ traces for each sample (Fig. S7). The chromatograms show a gradual decrease in the monomeric JSR1 peak at 2.8 min at elevated temperatures and indicate the production of oligomers at 60°C, followed by aggregation from 70°C onward (Fig. S7). To determine the melting points, each $UV_{280\ nm}$ absorption was plotted against the corresponding temperature, and the data were fitted with a Boltzmann sigmoidal function (Fig. 5 *D*). We successfully measured the melting temperatures in the ground and activated states for all six JSR1 variants and used the 95% confidence interval of the fit to approximate the statistical significance of the data (Fig. 5 *E*). All proteins display similar melting temperatures between 50°C and 55°C in their ground states. The activated states appear to be slightly destabilized, except for JSR1 WT. The strongest destabilization between the two states was observed for Y126D$^{3.28}$. A higher number of data points, particularly near the melting temperature, would enhance the quality of the fit and reveal clearer differences.

Utilizing the HPLC-DAD detector, we extracted UV/Vis spectra of each curve at 2.8 min to monitor the thermal degradation of λ_{max} (Fig. S8) followed by the same analysis as described above (Fig. S9 *A* and *B*). The results are in good agreement with melting points retrieved from $UV_{280\ nm}$ absorbance (Fig. S9 *C*). However, we observed a small systematic bias toward higher melting temperatures at $UV_{280\ nm}$. This may indicate that photoactivity is lost slightly before structural integrity is compromised.

Collectively, the established workflows enabled us to produce comprehensive insights into the expressibility and functionality of a large set of JSR1 mutants in a short time. The high-quality UV/Vis spectra from small amounts of affinity-purified protein facilitated the identification of five new mutants (S199Y$^{45.49}$, S199L$^{45.49}$, S199H$^{45.49}$, S199V$^{45.49}$, and S199I$^{45.49}$) exhibiting a similar behavior to the known S199F$^{45.49}$ mutation. Moreover, we found five additional variants (S199A$^{45.49}$, S199C$^{45.49}$, S199M$^{45.49}$, S199D$^{45.49}$, and S199E$^{45.49}$) that might function in a similar fashion under different experimental conditions. These 10 mutants are interesting targets for further upscaling, with a clear priority for the first five. They should be subjected to a more thorough characterization, gaining deeper insights into the underlying mechanism and their potential use in optogenetic applications.

Moreover, we could demonstrate for a small subset of mutants that the amounts of purified protein yielded from 25-mL cultures are sufficient to perform several other functional assays, providing additional insights such as G-protein activation and thermal stability in various activation states.

DISCUSSION

Miniaturized screening platform for the functional characterization of GPCRs

Integral membrane proteins, such as GPCRs, are notoriously challenging to work with due to their low expression yields (6,7), conformational flexibility (41), and poor stability outside their native membrane environment (42). These limitations often render wild-type GPCRs inaccessible for detailed structural and functional studies. Efficient variant screening and in vitro characterization are therefore essential to identify stable and expressible constructs for downstream applications.

Insect cell systems are widely used to express functionally folded membrane proteins, as they provide more advanced folding machinery and can perform eukaryotic posttranslational modifications, unlike *E. coli*. At the same time, they offer good scalability for protein production (5–9). However, the cumbersome high-titer baculovirus production procedure for many constructs in parallel and the need for careful culture conditions, such as high aeration and low shear stress, typically reduce their compatibility with miniaturized high-throughput formats (43,44). To overcome these limitations, we developed a highly efficient, small-scale workflow that enables parallel processing of 96 samples. We optimized and streamlined several steps of the baculovirus-insect cell workflow to establish a multiscale platform for rapid screening of large GPCR variant sets. Our system uses 96-well DWPs throughout virus production and expression screening, significantly increasing throughput over the commonly used 24-well format (43–49). Kärkkäinen et al. (50) demonstrated high-throughput virus production and titration 96-well DWPs, though using the Bac-to-Bac system. This approach requires a laborious, multistep bacmid preparation for each construct. In contrast, we employ selective homologous recombination (37,43) (e.g., *flashBAC*, Oxford Expression Technologies) for a simplified procedure. This method eliminates the need for individual bacmid preparations, reducing cost, time, and effort. Moreover, we have optimized the transfection conditions, reducing the time from 7 to 4 days (Fig. S3). This is essential in miniaturized suspension cultures, as every additional day increases evaporation, causing altered media conditions that impact cell fitness and viability (44).

Importantly, most previous approaches rely on SDS-PAGE or immunoblotting to assess protein expression, which can be labor intensive and only semiquantitative. Moreover, due to the denaturing conditions of such an analysis, they do not provide information on functional

expression or stability outside of the lipid bilayer. In contrast, our implementation of FSEC (14,15) enables rapid, automated evaluation of expression levels and protein quality under conditions preserving the protein's native fold. Therefore, we can assess the level of properly folded protein and the oligomerization and aggregation behavior (Fig. 3). This provides an early functional readout, facilitating rational variant selection.

However, low-expressing mutants might also be highly relevant for functional characterization. Hence, expression level alone might be an insufficient metric for variant prioritization. Structural and spectroscopic studies often require milligram quantities of purified protein, necessitating liter-scale cultures—an impractical approach for screening dozens of variants. To bridge this gap, we identified 25-mL cultures in TPP TubeSpin 50 Bioreactors as an ideal intermediate scale: large enough for functional assays, yet small enough for multiplexing.

As a first functional assay, we investigated the spectral absorption characteristics of our receptors, which present a hallmark feature of visual pigment functionality. We probed different ways of recording UV/Vis spectra of receptor variants purified from the 25-mL cultures. The spectra from benchtop spectrophotometers were often noisy and inconsistent due to varying purity and yields (Fig. S5). Ultimately, analytical size-exclusion chromatography with DAD provided the best results (Figs. 4 and S5). This method allowed separation of contaminants and real-time recording of high-quality absorption spectra from small amounts of purified protein. This significantly improved data comparability and purity assessment. It enabled us, in a single step, to confirm correct folding and chromophore uptake. Likely, due to the exceptional purity of the protein used for spectral analysis, the data allow estimation of extinction coefficients of each variant.

Although our approach is ideally suited for retinal-bound opsins, we explored its wider applicability by testing selected mutants in general GPCR assays. Functional readouts, such as G-protein activation and thermal stability, were feasible at a 25-mL scale (Fig. 5). Specifically, we adapted a thermal stability assay (40) using analytical size-exclusion chromatography to track protein unfolding by monitoring the degradation of the $UV_{280\ nm}$ absorbance upon heating (Fig. S7). This method requires neither an HPLC-DAD detector nor the presence of a chromophore. However, photoactive proteins might greatly benefit from a DAD detector as it allows the same analysis on the chromophore level (Figs. S8 and S9). For example, comparison of the melting points retrieved from $UV_{280\ nm}$ and λ_{max} (Fig. S9 *C*) might show the heat-induced chromophore loss before the protein unfolds. Following λ_{max} degradation could also be used to investigate temperature-induced chromophore isomerization. Moreover, the protein yields achieved at intermediate scale are sufficient for biophysical kinetic binding assays such as surface plasmon resonance spectroscopy or grating-coupled interferometry. Immobilization of membrane proteins for such assays typically requires nanomolar concentrations (51). Hence, our platform could support screening for GPCR-G-protein or ligand-binding kinetics. Additionally, it could facilitate the identification of stable complexes suitable for cryo-EM (52). Furthermore, fine-tuning of several baculovirus concentrations for optimal co-expression of the target GPCR with the G-protein subunits could be easily achieved (44,53).

The entire procedure up to the FSEC screening is carried out in 96-well plates and is highly compatible with automation; the only step not readily automatable is the manual loading and unloading of the ultracentrifuge rotor before the FSEC runs. However, since liquid handling systems are often not accessible to academic laboratories, the protocols were intentionally designed for use with handheld multichannel pipettes, enabling broad adoption without the need for specialized equipment. In contrast, the intermediate-scale characterization procedures are not yet compatible with automation and currently allow only for a modest degree of multiplexing. Future studies could aim to increase the throughput by employing ultracentrifuge rotors with higher tube capacity and automated purifications using magnetic beads in 24-well plates. Nonetheless, the described protocols already present a substantial improvement in both efficiency and throughput. A single researcher could complete the entire screening pipeline for 96 samples—from transfection to functional characterization—within 6 weeks, highlighting the platform's practicality and efficiency.

Obtaining multiscale functional insights across a large set of constructs within a short time frame represents a major improvement over conventional approaches and substantially rationalizes the selection of the most promising variants. Notably, the platform enables straightforward upscaling to preparative volumes following construct selection (Fig. 1). The V_0 and V_1 generation viruses produced in 96-well plates can be readily amplified at a larger scale. Specifically, up to 100 mL of fresh V_1 virus can be generated and stored in aliquots at $-80°C$, supporting the production of up to 10 L of high-titer V_2 virus. These volumes are typically sufficient for protein expression in several hundred liters. As a result, a single small-scale transfection often yields enough viral material to support an entire structural or functional study over several years.

Utilizing the platform to investigate bistability and color tuning of JSR1 mutants

To demonstrate the platform's capabilities, we screened 56 variants of the light-sensitive GPCR JSR1, focusing on the two residues $Y126^{3.28}$ and $S199^{45.49}$ that are located near the SB in the retinal binding pocket (26,28,29,54). Interestingly, $S199^{45.49}$ has been identified as a target residue for

color-tuning. It connects the distal counterion to the SB via a water-mediated hydrogen bond (Fig. 2 *A*). Substitution of S199$^{45.49}$ by phenylalanine disrupts the water-mediated hydrogen bond network and leads to a deprotonated SB in the dark state, yielding a distinct absorption profile and highlighting the functional relevance of single-residue substitutions in tuning spectral properties (26). Y126$^{3.28}$ occupies the position of the proximal counterion found in vertebrate opsins. It interacts directly with the SB and participates in the hydrogen bond network that connects to the distal counterion. Therefore, this residue is of particular interest for color-tuning mutations. In addition, we also hypothesized that it contributes to bistability, underscoring the importance of a functional characterization.

All constructs yielded viable virus stocks (Fig. 2 *B*). Expression screening revealed that substitutions at S199$^{45.49}$ were poorly tolerated, whereas many Y126$^{3.28}$ variants expressed at levels comparable to wild-type JSR1 (Fig. 3 *B* and *C*). Nevertheless, both sets of mutants are very interesting for studying the active site of this GPCR.

Using the high-titer virus stocks from the 96-well DWPs, we expressed >40 variants at this scale. A 2-day mini-purification workflow (Fig. S4) enabled us to reconstitute opsins with retinal and perform affinity purification for UV/Vis spectral analysis. Essential for purity, protein stability, and removal of all tags was the proteolytic elution under mild conditions from the affinity column. We could handle six samples per day, with one JSR1 WT control included to monitor for light contamination, allowing characterization of 20 variants per week. We purified 30 JSR1 variants and obtained a complete UV/Vis spectroscopy data set for the 9-*cis* retinal ground state. With simple illumination steps and subsequent HPLC analysis, we could also evaluate the spectra of the activated states (Fig. 4 *D*). The analysis of all spectra corroborated the FSEC findings on folding. Y126$^{3.28}$ mutations had modest effects on retinal absorption, whereas many S199$^{45.49}$ substitutions caused pronounced shifts. Ten variants exhibited altered color tuning, including the previously characterized S199F$^{45.49}$ mutant (26). Five previously uncharacterized variants, namely S199Y$^{45.49}$, S199L$^{45.49}$, S199H$^{45.49}$, S199V$^{45.49}$ and S199I$^{45.49}$ (Fig. 4 *D*), showed separated absorption spectra of the inactive and active state, which is most likely due to the deprotonation of the SB. These five mutants are very interesting for follow-up studies, including biophysics, structural analysis, or time-resolved spectroscopy to investigate bistability and color tuning in more detail. Specifically, the detailed absorption properties inside living cells should be explored as they might be slightly altered in a lipid environment compared with their detergent-solubilized form. This is important because these variants could also be candidates for optogenetic applications, which require precise information on spectral absorption profiles for photo switching between active and inactive states under native-like conditions.

Interestingly, we did not observe SB hydrolysis in any of the mutants, including those carrying the proximal counterion mutation Y126E$^{3.28}$ found in monostable opsins. This result contradicts earlier speculations that such substitutions might disrupt bistability. Instead, our findings suggest that bistability is not governed by a single residue but is more likely determined by the overall architecture of the retinal binding pocket.

Importantly, our screening results are in good agreement with previously characterized JSR1 variants. The wild-type JSR1, S199A, and S199F variants, all of which were previously expressed in HEK cells (24,28), were also expressed in our baculovirus system at levels sufficient for purification and showed the expected spectral properties. These findings validate the reliability of our screening approach. In contrast, the S199N variant, although previously published (26), did not express under our conditions in the baculovirus system. This discrepancy emphasizes the influence of subtle changes on expression behavior in different expression systems and highlights the value of systematic expression screening preceding large-scale characterization.

CONCLUSION

In summary, the scalable and modular nature of our platform enables rapid, information-rich screening of GPCR variants that is broadly applicable across multiple research domains. The screening platform is particularly valuable at the beginning of projects to identify the most promising variants, including protein homologs, truncated proteins, protein chimeras, differently tagged proteins, or point mutants. Beyond advancing our understanding of color-tuning and bistability in visual pigments, the workflow facilitates the identification of novel optogenetic tools and engineered opsin/GPCR chimeras with tailored properties. Furthermore, the platform enables the analysis of receptor expression, spectral properties, stability, and functional responses. This comprehensive approach can provide valuable insights into key aspects of GPCR biology, including ligand binding, activation mechanisms, and G-protein selectivity and specificity. These capabilities make it a powerful asset not only for fundamental GPCR research but also for accelerating drug discovery targeting this diverse and medically relevant receptor family. In addition, we foresee a more general applicability to other membrane protein targets.

ACKNOWLEDGMENTS

This project has received funding from the European Research Council (ERC) under the European Union's Horizon (H2020-EU.1.1. – EXCELLENT SCIENCE) research and innovation program ("ERC SOL," Synergy grant agreement ID: 951644). The grant funded the HPLC system. We thank Polina Isaikina, Xavier Deupi, and Francisco Leisico for valuable input and critical proofreading of the manuscript draft.

AUTHOR CONTRIBUTIONS

G.F.X.S. designed the project. J.M, D.W., and M.G. performed experiments. J.M. analyzed the data. J.M. and G.F.X.S. wrote the paper with input from all authors.

DECLARATION OF INTERESTS

G.F.X.S. is a co-founder and scientific advisor of leadXpro AG and InterAx Biotech AG. G.F.X.S.is serving as a guest editor of the special issue "Retinal Proteins: Experiment and Computation."

DECLARATION OF GENERATIVE AI AND AI-ASSISTED TECHNOLOGIES IN THE WRITING PROCESS

During the preparation of this work, the authors used ChatGPT (GPT-4o, OpenAI) in order to improve the grammar and readability. After using this tool/service, the authors reviewed and edited the content as needed and take full responsibility for the content of the publication.

SUPPORTING MATERIAL

Supporting material can be found online at https://doi.org/10.1016/j.bpj.2025.10.034.

REFERENCES

1. von Heijne, G. 2007. The membrane protein universe: what's out there and why bother? *J. Intern. Med.* 261:543–557. https://doi.org/10.1111/j.1365-2796.2007.01792.x.
2. Rosenbaum, D. M., S. G. F. Rasmussen, and B. K. Kobilka. 2009. The structure and function of G-protein-coupled receptors. *Nature.* 459:356–363. https://doi.org/10.1038/nature08144.
3. Venkatakrishnan, A. J., X. Deupi, …, M. M. Babu. 2013. Molecular signatures of G-protein-coupled receptors. *Nature.* 494:185–194. https://doi.org/10.1038/nature11896.
4. Hauser, A. S., M. M. Attwood, …, D. E. Gloriam. 2017. Trends in GPCR drug discovery: new agents, targets and indications. *Nat. Rev. Drug Discov.* 16:829–842. https://doi.org/10.1038/nrd.2017.178.
5. Wiseman, D. N., A. Otchere, …, A. D. Goddard. 2020. Expression and purification of recombinant G protein-coupled receptors: A review. *Protein Expr. Purif.* 167:105524. https://doi.org/10.1016/j.pep.2019.105524.
6. Schertler, G. F. X. 1992. Overproduction of membrane proteins. *Curr. Opin. Struct. Biol.* 2:534–544. https://doi.org/10.1016/0959-440X(92)90083-J.
7. Tate, C. G. 2001. Overexpression of mammalian integral membrane proteins for structural studies. *FEBS Lett.* 504:94–98. https://doi.org/10.1016/s0014-5793(01)02711-9.
8. Massotte, D. 2003. G protein-coupled receptor overexpression with the baculovirus-insect cell system: a tool for structural and functional studies. *Biochim. Biophys. Acta.* 1610:77–89. https://doi.org/10.1016/s0005-2736(02)00720-4.
9. Lv, X., J. Liu, …, R. C. Stevens. 2016. In vitro expression and analysis of the 826 human G protein-coupled receptors. *Protein Cell.* 7:325–337. https://doi.org/10.1007/s13238-016-0263-8.
10. Tate, C. G., and G. F. X. Schertler. 2009. Engineering G protein-coupled receptors to facilitate their structure determination. *Curr. Opin. Struct. Biol.* 19:386–395. https://doi.org/10.1016/j.sbi.2009.07.004.
11. Rosenbaum, D. M., V. Cherezov, …, B. K. Kobilka. 2007. GPCR engineering yields high-resolution structural insights into beta2-adrenergic receptor function. *Science.* 318:1266–1273. https://doi.org/10.1126/science.1150609.
12. Chun, E., A. A. Thompson, …, R. C. Stevens. 2012. Fusion partner toolchest for the stabilization and crystallization of G protein-coupled receptors. *Structure.* 20:967–976. https://doi.org/10.1016/j.str.2012.04.010.
13. Jaeger, K., S. Bruenle, …, J. Standfuss. 2019. Structural Basis for Allosteric Ligand Recognition in the Human CC Chemokine Receptor 7. *Cell.* 178:1222–1230.e10. https://doi.org/10.1016/j.cell.2019.07.028.
14. Kawate, T., and E. Gouaux. 2006. Fluorescence-detection size-exclusion chromatography for precrystallization screening of integral membrane proteins. *Structure.* 14:673–681. https://doi.org/10.1016/j.str.2006.01.013.
15. Shiroishi, M., H. Tsujimoto, …, T. Kobayashi. 2012. Platform for the rapid construction and evaluation of GPCRs for crystallography in Saccharomyces cerevisiae. *Microb. Cell Fact.* 11:78. https://doi.org/10.1186/1475-2859-11-78.
16. Terakita, A. 2005. The opsins. *Genome Biol.* 6:213. https://doi.org/10.1186/gb-2005-6-3-213.
17. Ernst, O. P., D. T. Lodowski, …, H. Kandori. 2014. Microbial and animal rhodopsins: structures, functions, and molecular mechanisms. *Chem. Rev.* 114:126–163. https://doi.org/10.1021/cr4003769.
18. Isberg, V., C. de Graaf, …, D. E. Gloriam. 2015. Generic GPCR residue numbers - aligning topology maps while minding the gaps. *Trends Pharmacol. Sci.* 36:22–31. https://doi.org/10.1016/j.tips.2014.11.001.
19. Abreu, N., and J. Levitz. 2020. Optogenetic Techniques for Manipulating and Sensing G Protein-Coupled Receptor Signaling. *Methods Mol. Biol.* 2173:21–51. https://doi.org/10.1007/978-1-0716-0755-8_2.
20. Deisseroth, K. 2011. Optogenetics. *Nat. Methods.* 8:26–29. https://doi.org/10.1038/nmeth.f.324.
21. Lehtinen, K., M. S. Nokia, and H. Takala. 2021. Red Light Optogenetics in Neuroscience. *Front. Cell. Neurosci.* 15:778900. https://doi.org/10.3389/fncel.2021.778900.
22. Porter, M. L., H. Awata, …, T. W. Cronin. 2020. Exceptional diversity of opsin expression patterns in Neogonodactylus oerstedii (Stomatopoda) retinas. *Proc. Natl. Acad. Sci. USA.* 117:8948–8957. https://doi.org/10.1073/pnas.1917303117.
23. Wang, Z., A. B. Asenjo, and D. D. Oprian. 1993. Identification of the Cl(-)-binding site in the human red and green color vision pigments. *Biochemistry.* 32:2125–2130. https://doi.org/10.1021/bi00060a001.
24. Asenjo, A. B., J. Rim, and D. D. Oprian. 1994. Molecular determinants of human red/green color discrimination. *Neuron.* 12:1131–1138. https://doi.org/10.1016/0896-6273(94)90320-4.
25. Wang, W., Z. Nossoni, …, B. Borhan. 2012. Tuning the electronic absorption of protein-embedded all-trans-retinal. *Science.* 338:1340–1343. https://doi.org/10.1126/science.1226135.
26. Nagata, T., M. Koyanagi, …, A. Terakita. 2019. The counterion-retinylidene Schiff base interaction of an invertebrate rhodopsin rearranges upon light activation. *Commun. Biol.* 2:180. https://doi.org/10.1038/s42003-019-0409-3.
27. Nagata, T., M. Koyanagi, …, A. Terakita. 2012. Depth perception from image defocus in a jumping spider. *Science.* 335:469–471. https://doi.org/10.1126/science.1211667.
28. Varma, N., E. Mutt, …, E. Lesca. 2019. Crystal structure of jumping spider rhodopsin-1 as a light sensitive GPCR. *Proc. Natl. Acad. Sci. USA.* 116:14547–14556. https://doi.org/10.1073/pnas.1902192116.
29. Tejero, O., F. Pamula, …, C. J. Tsai. 2024. Active state structures of a bistable visual opsin bound to G proteins. *Nat. Commun.* 15:8928. https://doi.org/10.1038/s41467-024-53208-2.
30. Ehrenberg, D., N. Varma, …, E. Lesca. 2019. The Two-Photon Reversible Reaction of the Bistable Jumping Spider Rhodopsin-1. *Biophys. J.* 116:1248–1258. https://doi.org/10.1016/j.bpj.2019.02.025.
31. Abdulrahman, W., M. Uhring, …, A. Poterszman. 2009. A set of baculovirus transfer vectors for screening of affinity tags and parallel expression strategies. *Anal. Biochem.* 385:383–385. https://doi.org/10.1016/j.ab.2008.10.044.

32. Kubala, M. H., O. Kovtun, …, B. M. Collins. 2010. Structural and thermodynamic analysis of the GFP:GFP-nanobody complex. *Protein Sci.* 19:2389–2401. https://doi.org/10.1002/pro.519.

33. Rodrigues, M. J., O. Tejero, …, G. F. X. Schertler. 2024. Activating an invertebrate bistable opsin with the all-trans 6.11 retinal analog. *Proc. Natl. Acad. Sci. USA.* 121:e2406814121. https://doi.org/10.1073/pnas.2406814121.

34. Mühle, J., J. Alenfelder, …, X. Deupi. 2025. Cyclic peptide inhibitors function as molecular glues to stabilize Gq/11 heterotrimers. *Proc. Natl. Acad. Sci. USA.* 122:e2418398122. https://doi.org/10.1073/pnas.2418398122.

35. 2020. EnzyScreen. https://enzyscreen.com/article/hydrodynamicsotr-recommendations-and-background/?utm_source=chatgpt.com.

36. Duetz, W. A., L. Rüedi, …, B. Witholt. 2000. Methods for intense aeration, growth, storage, and replication of bacterial strains in microtiter plates. *Appl. Environ. Microbiol.* 66:2641–2646. https://doi.org/10.1128/AEM.66.6.2641-2646.2000.

37. Zhao, Y., D. A. G. Chapman, and I. M. Jones. 2003. Improving baculovirus recombination. *Nucleic Acids Res.* 31:E6-6. https://doi.org/10.1093/nar/gng006.

38. Zhang, Z., X. Zhang, and H. Chen. 2024. TCID(50) Assay: A Simple Method to Determine Baculovirus Titer. *Methods Mol. Biol.* 2829:267–270. https://doi.org/10.1007/978-1-0716-3961-0_20.

39. Ahuja, S., M. Eilers, …, S. O. Smith. 2009. 6-s-cis Conformation and polar binding pocket of the retinal chromophore in the photoactivated state of rhodopsin. *J. Am. Chem. Soc.* 131:15160–15169. https://doi.org/10.1021/ja9034768.

40. Hattori, M., R. E. Hibbs, and E. Gouaux. 2012. A fluorescence-detection size-exclusion chromatography-based thermostability assay for membrane protein precrystallization screening. *Structure.* 20:1293–1299. https://doi.org/10.1016/j.str.2012.06.009.

41. Kobilka, B. K., and X. Deupi. 2007. Conformational complexity of G-protein-coupled receptors. *Trends Pharmacol. Sci.* 28:397–406. https://doi.org/10.1016/j.tips.2007.06.003.

42. Magnani, F., Y. Shibata, …, C. G. Tate. 2008. Co-evolving stability and conformational homogeneity of the human adenosine A2a receptor. *Proc. Natl. Acad. Sci. USA.* 105:10744–10749. https://doi.org/10.1073/pnas.0804396105.

43. Possee, R. D., R. B. Hitchman, …, L. A. King. 2008. Generation of baculovirus vectors for the high-throughput production of proteins in insect cells. *Biotechnol. Bioeng.* 101:1115–1122. https://doi.org/10.1002/bit.22002.

44. Bahia, D., R. Cheung, …, I. Hunt. 2005. Optimisation of insect cell growth in deep-well blocks: development of a high-throughput insect cell expression screen. *Protein Expr. Purif.* 39:61–70. https://doi.org/10.1016/j.pep.2004.08.020.

45. Buchs, M., E. Kim, …, I. Hunt. 2009. High-throughput insect cell protein expression applications. *Methods Mol. Biol.* 498:199–227. https://doi.org/10.1007/978-1-59745-196-3_14.

46. Kalathur, R. C., M. Panganiban, and R. Bruni. 2016. High-Throughput Baculovirus Expression System for Membrane Protein Production. *Methods Mol. Biol.* 1432:187–202. https://doi.org/10.1007/978-1-4939-3637-3_12.

47. Hitchman, R. B., R. D. Possee, and L. A. King. 2012. High-throughput baculovirus expression in insect cells. *Methods Mol. Biol.* 824:609–627. https://doi.org/10.1007/978-1-61779-433-9_33.

48. McCall, E. J., A. Danielsson, …, W. M. Abbott. 2005. Improvements to the throughput of recombinant protein expression in the baculovirus/insect cell system. *Protein Expr. Purif.* 42:29–36. https://doi.org/10.1016/j.pep.2005.03.021.

49. Shrestha, B., C. Smee, and O. Gileadi. 2008. Baculovirus expression vector system: an emerging host for high-throughput eukaryotic protein expression. *Methods Mol. Biol.* 439:269–289. https://doi.org/10.1007/978-1-59745-188-8_19.

50. Karkkainen, H. R., H. P. Lesch, …, S. Yla-Herttuala. 2009. A 96-well format for a high-throughput baculovirus generation, fast titering and recombinant protein production in insect and mammalian cells. *BMC Res. Notes.* 2:63. https://doi.org/10.1186/1756-0500-2-63.

51. Chu, R., D. Reczek, and W. Brondyk. 2014. Capture-stabilize approach for membrane protein SPR assays. *Sci. Rep.* 4:7360. https://doi.org/10.1038/srep07360.

52. Pamula, F., J. Muhle, …, C. J. Tsai. 2020. Strategic Screening and Characterization of the Visual GPCR-mini-G Protein Signaling Complex for Successful Crystallization. *J. Vis. Exp.* 157:e60747. https://doi.org/10.3791/60747.

53. Liang, Y. L., M. Khoshouei, …, P. M. Sexton. 2017. Phase-plate cryo-EM structure of a class B GPCR-G-protein complex. *Nature.* 546:118–123. https://doi.org/10.1038/nature22327.

54. Koyanagi, M., T. Nagata, …, F. Tokunaga. 2008. Molecular evolution of arthropod color vision deduced from multiple opsin genes of jumping spiders. *J. Mol. Evol.* 66:130–137. https://doi.org/10.1007/s00239-008-9065-9.

Biophysical Journal, Volume *125*

Supplemental information

A multiscale screening platform for rapid GPCR variant profiling reveals color-tuned JSR1 mutants

Jonas Mühle, Deborah Walter, Marielouise Griebl, and Gebhard F.X. Schertler

A multiscale screening platform for rapid GPCR variant profiling reveals color-tuned JSR1 mutants

Supplemental material

Jonas Mühle[1,*], Deborah Walter[1,2], Marielouise Griebl[1,§], Gebhard F. X. Schertler[1,*]

1 Center for Life Sciences, Laboratory of Biomolecular Research, Paul Scherrer Institute, Villigen PSI, Switzerland.

2 Biomolecular Structure and Mechanism PhD Program of the Life Science Zurich Graduate School, University of Zurich, Switzerland

§ Current address: Molecular Biotechnology Master Program, Faculty for Engineering, Heidelberg University, Germany

* correspondence to: jonas.muehle@psi.ch and gebhard.schertler@psi.ch

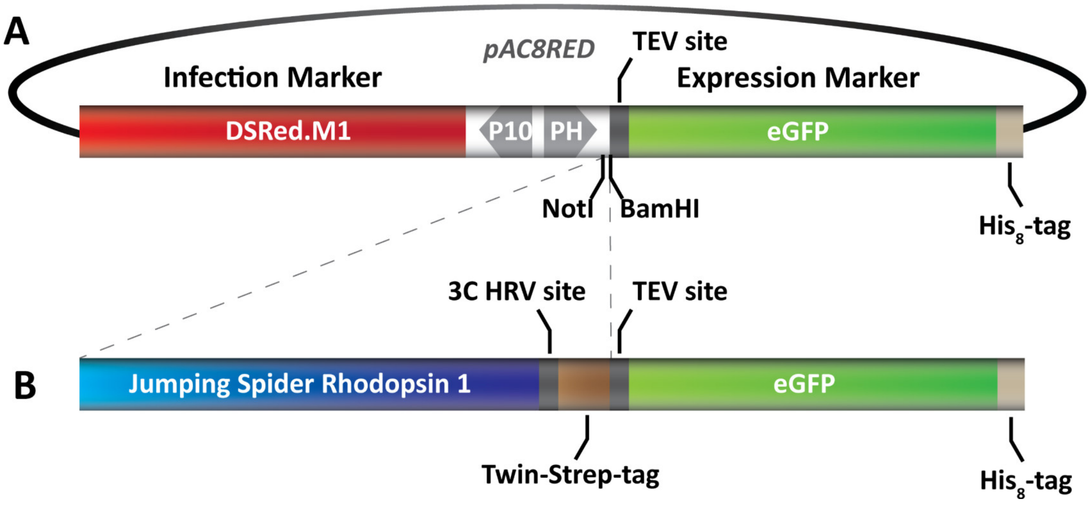

Supplemental Figure S1: Plasmid and construct design of the JSR1-eGFP fusions. A) *pAC8RED backbone with two fluorescent proteins. DsRed.M1 under the P10 promoter control used as an infection marker and TEV-eGFP-His$_8$ sequence under the PH promoter control. NotI and BamHI restriction sites are used to insert covalent fusions of the target proteins N-terminally to the TEV-eGFP-His$_8$ cassette.* ***B)*** *Jumping Spider Rhodopsin 1 (JSR1) inserted via the NotI/BamHI sites. A 3C HRC protease sequence followed by a Twin-Strep-tag was fused C-terminally to JSR1.*

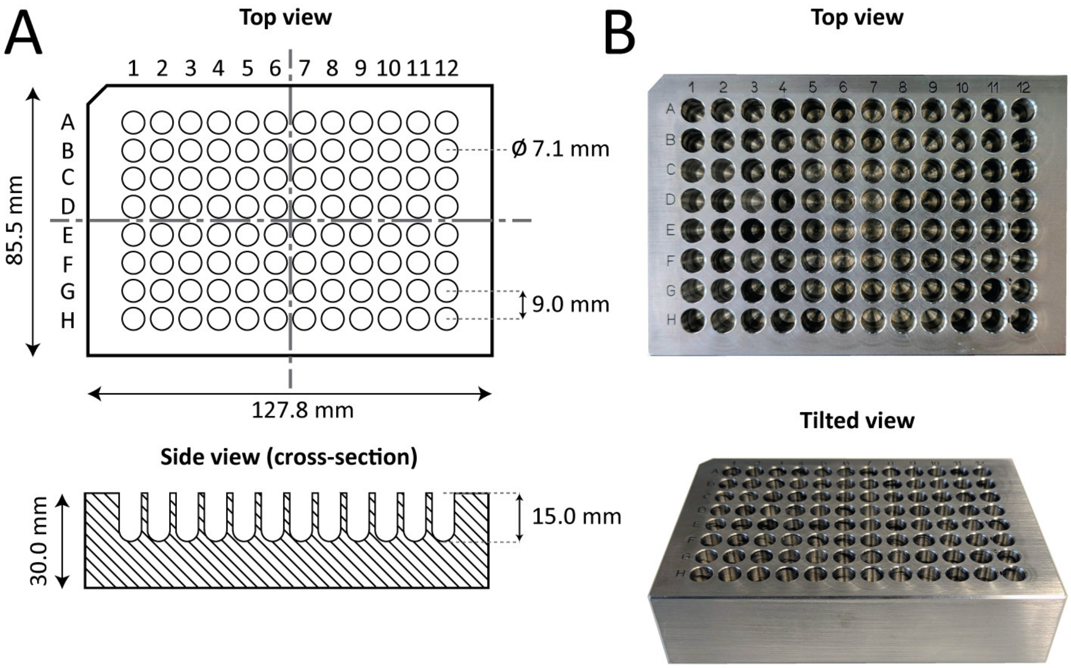

***Supplemental Figure S2: Blueprint and images of the custom-made ultracentrifuge tube holder for Beckmann Type 42.2 Ti tubes**. **A)** Top view and side view blueprint of the tube holder. The holder was manufactured from a solid aluminum block according to standard 96-well microplate dimensions defined by ANSI/SLAS (https://www.slas.org/SLAS/assets/File/public/standards/ANSI_SLAS_1-2004_FootprintDimensions.pdf). For efficient temperature control, the plate height is 30.0 mm and the holes are 15.0 mm deep with a 7.1 mm diameter. **B)** Top view and tilted view images of the tube holder. The metal block was not built for the use in any centrifuge or ultracentrifuge. It does not hold 96-well plates or enables high-speed centrifugations of those. It solely provides an efficient opportunity to transfer liquids from 96-well plates into single ultracentrifuge tubes and vice versa using multichannel pipets before and after ultracentrifugation. It helps reduce pipetting errors or mix-up of samples (particularly in dim-light conditions) while keeping the samples cold (or warm, if desired).*

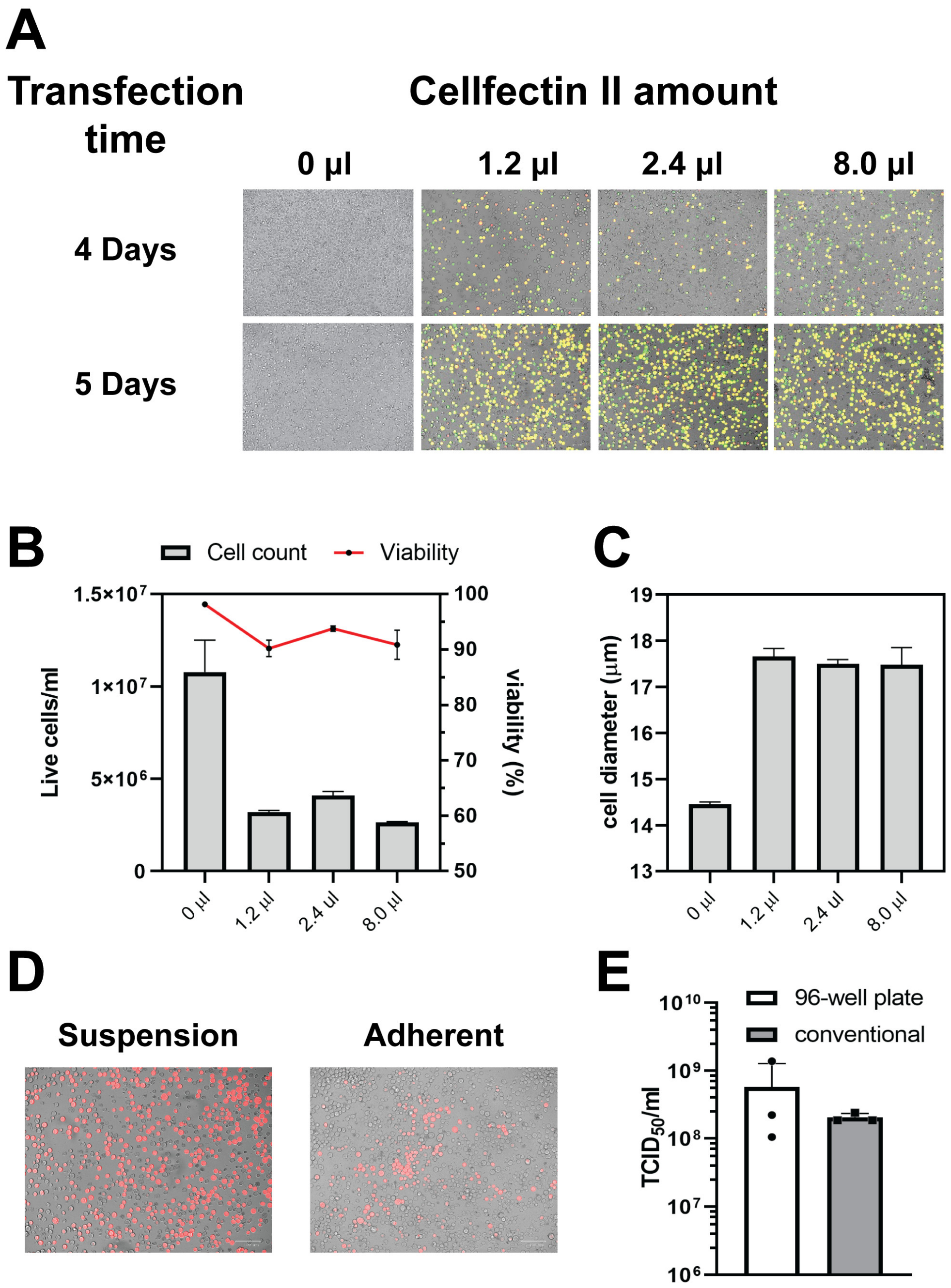

***Supplemental Figure S3**: **Transfection and virus amplification in suspension. A)** Transfection efficiency monitored by fluorescence microscopy after 4 and 5 days post-transfection at different Cellfectin II concentrations. **B) and C)** Cell fitness parameters (cell density, viability, and diameter) were assessed 4 days post-transfection. The sample names correspond to the Cellfectin II amounts used for transfection. All transfected cells show clear signs of a viral infection compared to the negative control ("0 µl"): reduced viability, reduced cell growth, and increased cell diameter. No significant differences between the three transfected samples are observed. Values are derived from two independent replicates, displayed ± S.D. **D)** Comparison of transfection in suspension (in 96-Well DWPs) with conventional protocols in adherent cell culture (in 6 well plates) monitored by fluorescence microscopy 5 days post-transfection. **E)** Assessment of viral strength after one cycle of virus amplification (V_1 virus stocks) in suspension cultures using a TCID50 assay. Both methods produce virus stocks with very similar viral strengths. Values represent the mean ± S.D. of three independent replicates.*

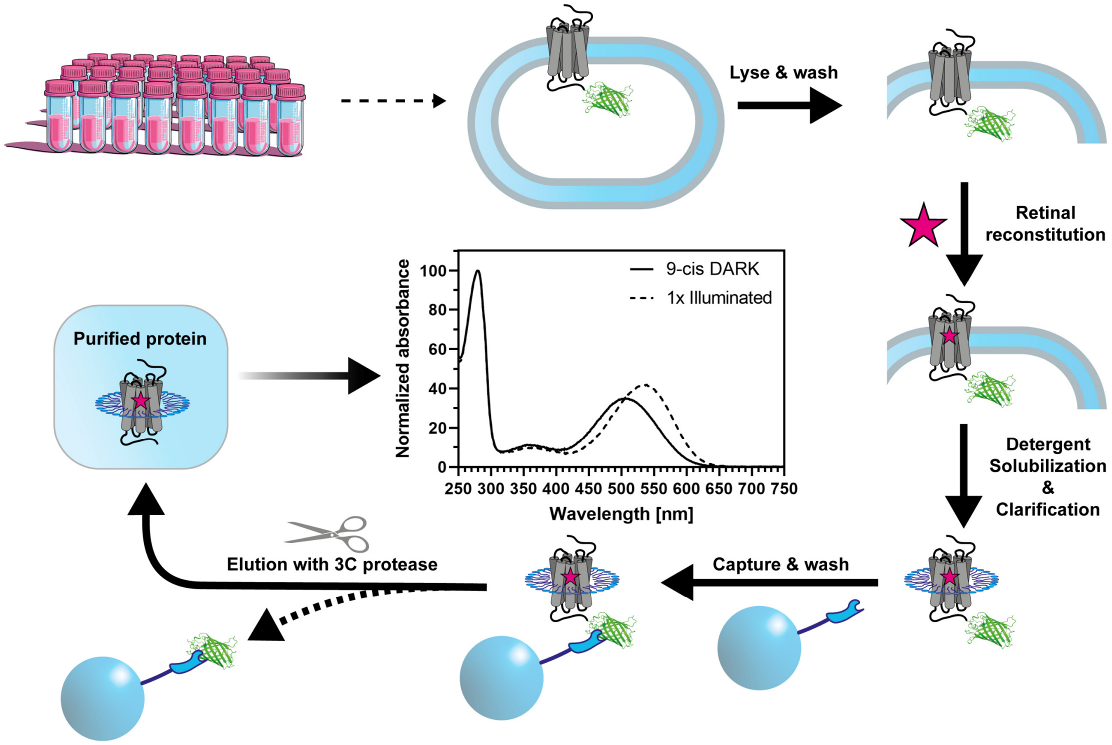

***Supplemental Figure S4**: **Workflow from 25 ml expression cultures to UV/Vis absorption analysis** of small-scale purified proteins. The proteins were expressed in TPP Tubespin Bioreactors 50. The pellets were lysed, and the insoluble fraction was washed before JSR1 reconstitution with retinal. The detergent-solubilized receptor was captured by anti-eGFP- or StrepTactin XT resin, washed, and eluted with 3C protease, which yielded highly pure, fluorophore-free JSR1 variants for the characterization of the UV/Vis absorption properties.*

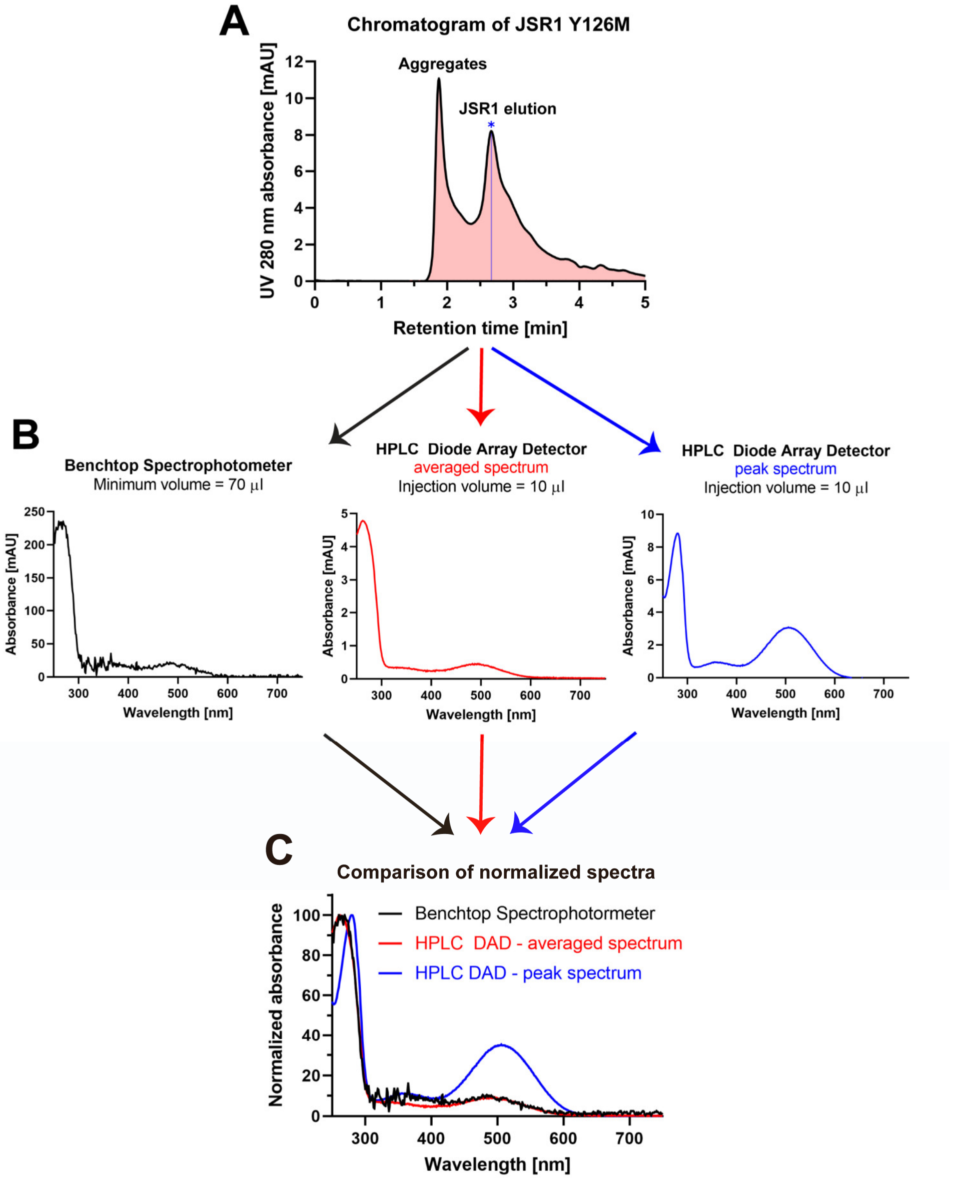

Supplemental Figure S5: Comparison of UV/Vis absorption spectra recorded on a benchtop spectrophotometer and HPLC Diode Array Detector (DAD). A) *Chromatogram of affinity-purified JSR1 Y126M. The sample contained a significant carryover of aggregates and was eluted in a total of 300 µl elution buffer due to technical complications during the small-scale purification procedure. The sample represents the most impure sample of the entire set of mutants and was used to compare the data quality of different absorption spectra acquisition methods.* ***B)*** *UV/Vis absorption spectra acquired from the purified sample. Left: Using a standard benchtop spectrophotometer and a cuvette with 10 mm pathlength. The spectrum displays a large peak at 260/280 nm, a noisy region between 300-400 nm, and a small chromophore absorption peak around 500 nm. Center: Spectrum retrieved from the HPLC DAD detector by averaging all spectra across the entire size exclusion chromatography run (red area in the chromatogram above). This spectrum simulates the spectrum from the benchtop spectrophotometer as it contains all components from the affinity purification without further size exclusion chromatography purification. Despite a 50 times lower signal intensity, the spectrum displays much less noise. Right: Peak spectrum extracted from the peak maximum of the JSR1 Y126M elution (indicated with a blue line and an asterisk in the chromatogram). The spectrum shows a clear peak at 280 nm and a strong chromophore absorption peak around 500 nm.* ***C)*** *Overlay of the three spectra after normalization to the global peak maximum. The averaged DAD spectrum is indeed highly similar to the benchtop spectrophotometer spectrum. However, the DAD provides a substantially improved signal stability across the entire spectral range. The DAD peak spectrum has a clearer 280 nm peak and a significantly increased 280 nm/ 500 nm ratio compared to the other spectra. This comparison highlights the power of an HPLC-DAD-based UV/Vis analysis over conventional methods. It drastically enhances the confidence of the analysis and eliminates the need for larger quantities of highly purified proteins.*

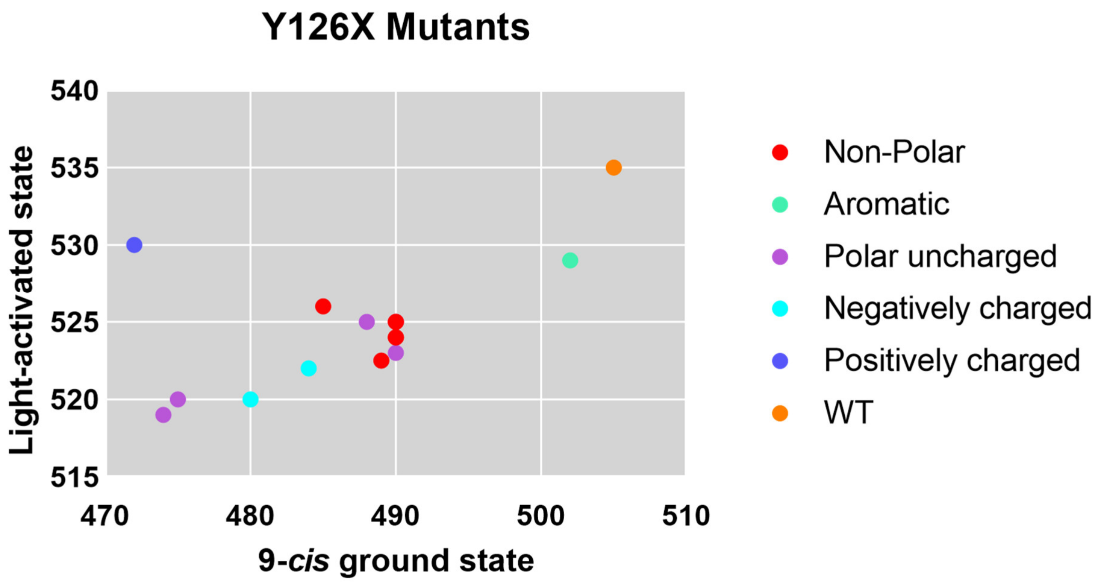

***Supplemental Figure S6**: **Analysis of λ_{max} of all Y126X mutants in the 9-cis dark state against the activated state**. The WT (Y126) is the most red-shifted variant at that position in both states. The overall most similar mutant in both states is Y126F (the only aromatic mutant that expressed). Y126H (the only positively charged mutant that expressed) induces the largest blue shift in the 9-cis dark state while retaining the most similar absorption to the WT in the active state, leading to an enlarged spectral separation of the two states.*

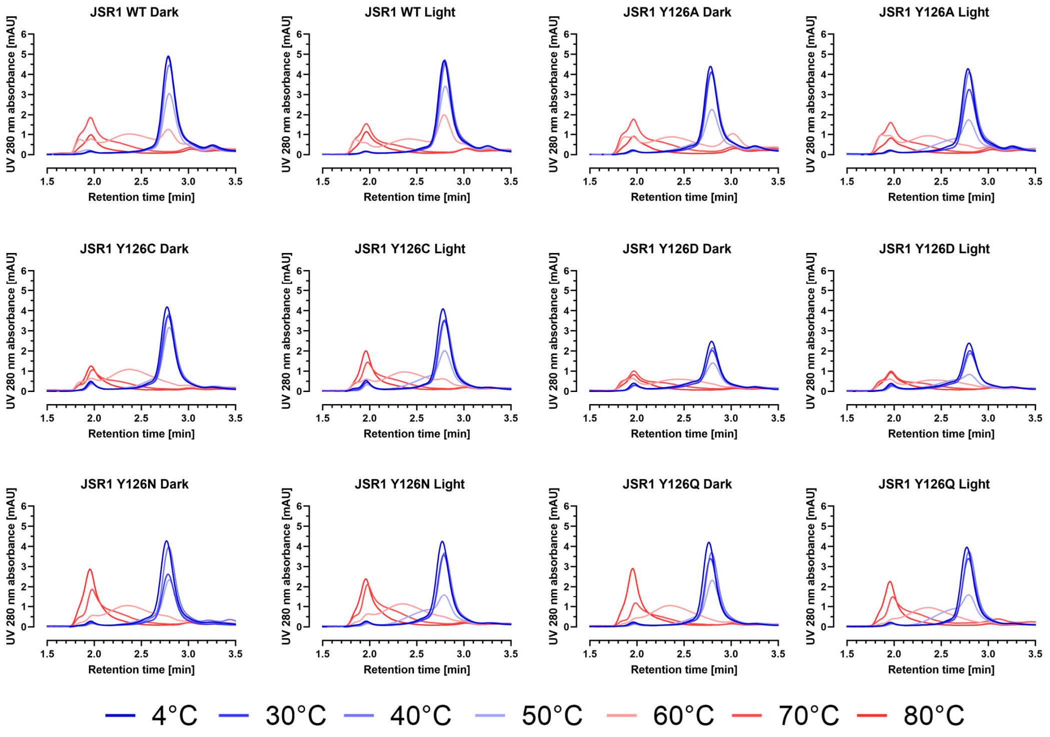

***Supplemental Figure S7**: **Analytical Size Exclusion chromatograms (UV$_{280nm}$) used to assess the thermal stability of JSR1 WT and five Y126X mutants**. Each panel displays all chromatograms from a single JSR1 variant in either the ground state ("Dark") or the light-activated state ("Light"). The samples were incubated for 5 minutes at different temperatures prior to the SEC analysis, and the chromatograms are colored according to the temperature gradient from blue (4°C) to red (80°C). UV/Vis spectra were extracted at the monomeric peak position of JSR1 at 2.8 minutes and plotted in Supplemental Figure S8.*

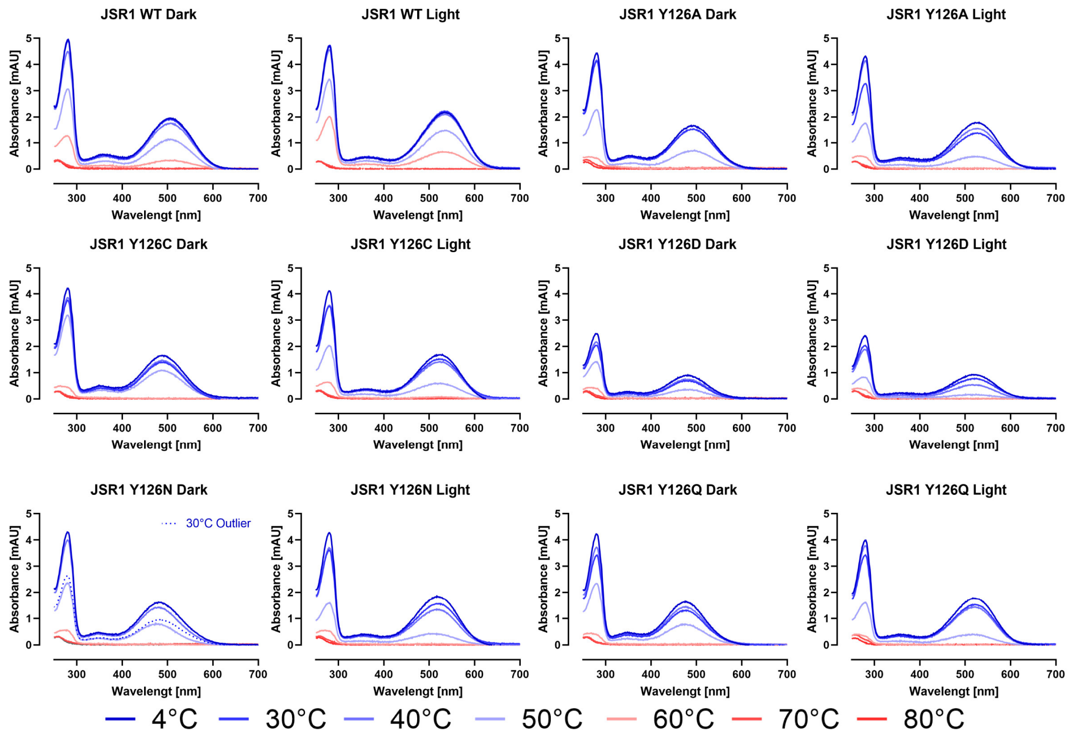

***Supplemental Figure S8**: **UV/Vis spectra extracted at 2.8 min from the UV_{280nm} chromatograms in Supplemental Figure S7**. Each panel displays all spectra from a single JSR1 variant in either the ground state ("Dark") or the light-activated state ("Light"). The samples were incubated for 5 minutes at different temperatures prior to the SEC analysis, and the spectra are colored according to the temperature gradient from blue (4°C) to red (80°C). We used the thermal degradation of the chromophore absorption peak to determine the melting temperatures (see Fig. S9).*

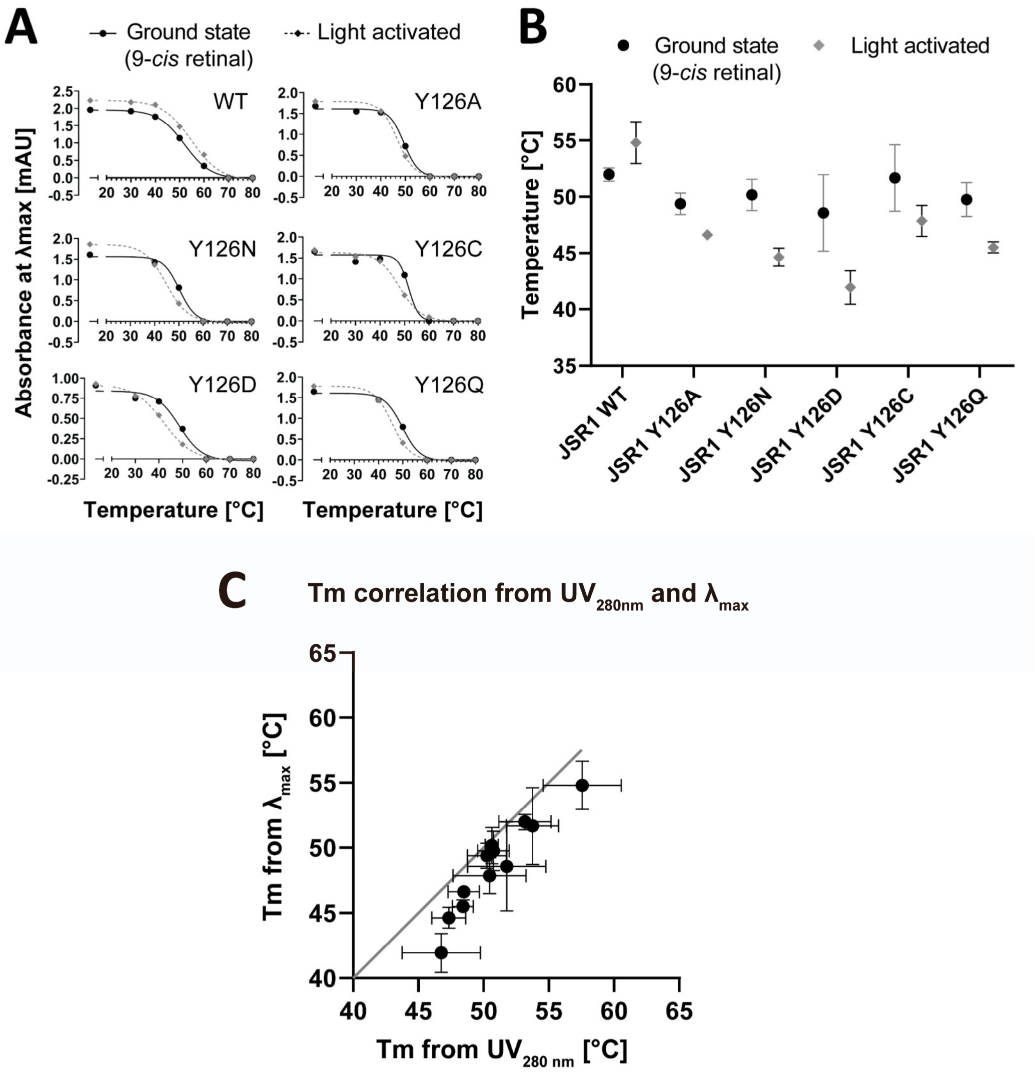

Supplemental Figure S9: **Thermostability data derived from the chromophore absorption. A)** *HPLC-derived melting curves of different JSR1 mutants obtained from the decay of the chromophore absorption at increasing temperatures (see Suppl. Fig. S8). The data represent the peak height of the chromophore absorption peak at the corresponding temperature. Values from the ground state proteins are shown as black dots, while data after light activation are shown as grey rhombuses. The data was fitted by a Boltzmann sigmoidal equation. Fitted curves are shown as black solid (ground state) and grey dashed (active state) lines.* ***B)*** *Melting temperatures calculated from the melting curves. The melting points are shown as black dots (ground state) and grey rhombuses (light-activated state). Error bars indicate the upper and lower limits of the 95% confidence interval of the fit.* ***C)*** *Scatter plot showing the correlation between the melting temperature (Tm) calculations from the UV_{280nm} signal (plotted on the X-axis) vs the λ_{max} of the JSR1 chromophore (plotted on the Y-axis). The data is shown as black dots with their corresponding upper and lower limits of the 95% confidence interval of the fit. Each point represents one sample. A diagonal through the origin with slope = 1 is shown as a grey line representing the theoretical perfect correlation. For most of the samples, this diagonal is within the 95% confidence interval, demonstrating a good agreement between both methods. However, a small systematic bias towards higher melting temperatures from the UV_{280nm} data is observed.*
